# One‐Pot Encapsulation of Enzymes in a Calcium Carboxylate Metal‐Organic Framework for Improved Buffer Stability

**DOI:** 10.1002/advs.202510960

**Published:** 2025-10-02

**Authors:** Ying Shu, Weibin Liang, Jun Huang

**Affiliations:** ^1^ School of Chemical and Biomolecular Engineering University of Sydney Darlington NSW 2008 Australia

**Keywords:** buffer stability, calcium carboxylate MOF, enzyme encapsulation, machine learning optimization, metal‐organic frameworks

## Abstract

Diverse metal‐organic frameworks (MOFs) have been actively studied for enzyme encapsulation to enhance encapsulation efficiency (EE), retained enzymatic activity (REA), and stability. This study develops a biocompatible synthesis protocol for a MOF constructed from Ca^2^⁺ and 4,5‐imidazoledicarboxylate ligand (termed CaIDC) in water at room temperature. This method enabled in situ, one‐pot encapsulation of various enzymes, including bovine serum albumin (BSA), glucose oxidase (GOx), catalase (CAT), and esterase (EST). Compared to widely studied zeolitic imidazolate frameworks (ZIFs), CaIDC‐based biocomposites demonstrated enhanced chemical robustness in phosphate buffer at pH 6.0 and 7.4. To optimize the synthesis process, a machine learning‐assisted workflow incorporating Latin hypercube sampling (LHS) is developed for an efficient exploration of the entire synthesis space. As a model system, the optimized EST@CaIDC sample (EC19) exhibited EE, REA, and protein loading (P_loading_) values of 28.7%, 20.1%, and 4.2 wt.%, respectively. In all, this study presents the development of a robust CaIDC‐based platform for enzyme encapsulation and the implementation of an efficient ML‐assisted optimization strategy, offering a pathway to advance enzyme encapsulation technologies with enhanced catalytic performance and sustainability.

## Graphical abstract

A biocompatible, room‐temperature synthesis of Ca^2^⁺–4,5‐imidazoledicarboxylate MOFs enables in situ encapsulation of diverse enzymes with enhanced stability in phosphate buffer. A machine learning workflow integrating Latin hypercube sampling efficiently navigates the synthesis space, optimizing encapsulation efficiency, retained enzymatic activity, and protein loading, establishing a robust and sustainable platform for advanced enzyme encapsulation technologies.

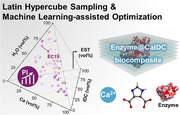

## 1 Introduction

Enzymatic reactions hold immense scientific and industrial importance in chemical production due to their high reactivity, efficiency, and stereoselectivity under mild conditions (ambient temperature, pressure, and pH).^[^ ^1^ ^]^ However, the practical applications of biocatalysis are hindered by enzymes’ sensitivity to environmental stressors, such as extreme pH, organic solvents, high temperatures, prolonged use, as well as poor recyclability.^[^ ^2^ ^]^ Enzyme immobilization offers a promising solution to address these challenges, enhancing the enzyme's adaptability under industrial operating conditions and improving recyclability for economic sustainability.^[^ ^3^ ^]^ Recently, metal‐organic frameworks (MOFs) have emerged as promising platforms for enzyme immobilization.^[^ ^4^ ^]^ Among them, zeolitic imidazolate frameworks (ZIFs) or metal azolate frameworks (MAFs) are widely studied due to their ability to be synthesized in water at room temperature.^[^ ^4a^ ^]^ Since the pioneering works reported by Ge,^[^ ^5^ ^]^ Tsung,^[^ ^6^ ^]^ and Falcaro,^[^ ^7^ ^]^ various ZIF‐based biocomposites have been developed using different ZIFs and enzyme molecules.^[^ ^4a^ ^]^ These ZIF‐based biocomposites have demonstrated significant potential in retaining enzymatic activity and improving enzyme stability. However, recent studies have demonstrated that ZIF‐based materials can gradually decompose in common biological buffers, e.g., phosphate buffer (PB).^[^ ^8^ ^]^ Efforts to improve ZIF stability through the introduction of additives (e.g., poly(glutamic acid)^[^ ^9^ ^]^) during biocomposite synthesis represent a promising research direction. To address this challenge from another angle, research has increasingly focused on exploring more chemically robust MOF platforms with stronger metal‐ligand coordinating bonds. These include frameworks built from hard metals and carboxylate ligands, such as UiO‐66,^[^ ^10^ ^]^ UiO‐66‐F4,^[^ ^11^ ^]^ Ca‐MOM,^[^ ^12^ ^]^ MIL‐101,^[^ ^13^ ^]^ MOF‐801,^[^ ^14^ ^]^ and NH_2_‐MIL‐53(Al).^[^ ^15^ ^]^ Despite the potential of these carboxylate MOFs in enzyme immobilization applications, their synthesis often requires organic solvents or extreme pH conditions to facilitate ligand dissolution and/or framework crystallization. Alternative stable reticular framework materials,^[^ ^4j^ ^]^ such as covalent organic frameworks (COFs)^[^ ^16^ ^]^ and hydrogen‐bonding organic frameworks (HOFs),^[^ ^17^ ^]^ are also being actively explored.

**Figure 1:**
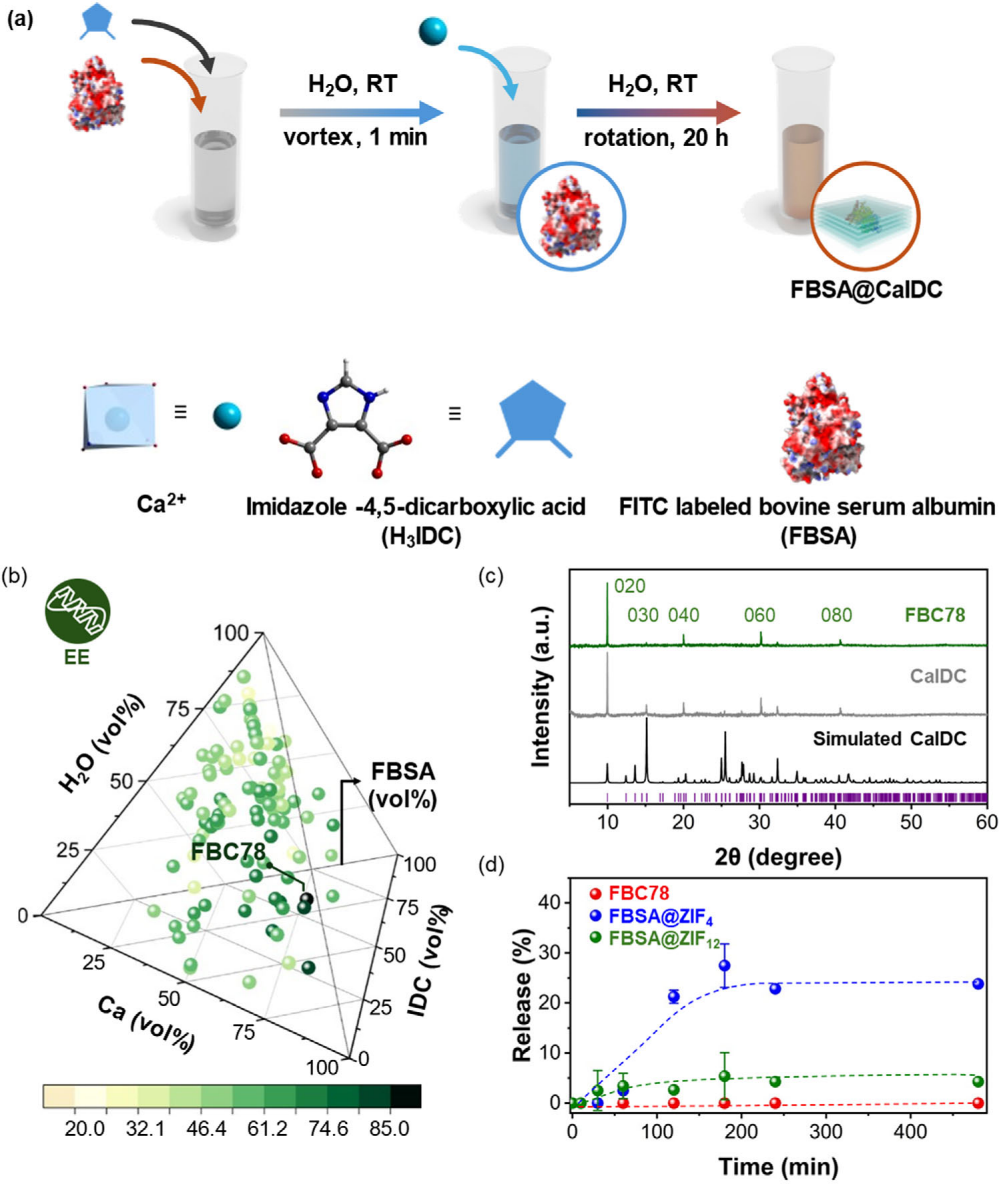
a) Schematic representation of the synthesis protocol for FBSA/CaIDC. b) 3D pyramid plot showing the EE values for all studied FBSA/CaIDC samples. The color scale represents the EE values (%) of each sample. c) PXRD patterns of simulated CaIDC (black), experimental CaIDC (grey), and FBC78 (green). d) Release kinetics of FBSA from FBC78 (red), FBSA@ZIF_4_ (blue), and FBSA@ZIF_12_ (green) in a pH 7.4 PB buffer over an 8‐h treatment.

In this study, we investigate the use of a MOF structure constructed by the Ca^2^⁺ and 4,5‐imidazolecarboxylate (IDC) ligand for enzyme immobilization, which is expected to possess higher chemical stability in buffer solutions as compared to ZIF‐based materials. Ca^2^⁺ was chosen for its strong coordination to carboxylate ligand, and IDC ligand was selected due to its moderate water solubility, strong coordination properties with hard metal,^[^ ^18^ ^]^ and versatile coordination modes toward Ca^2+^. MOF‐based biocomposites can be obtained using either one‐pot synthesis, post‐synthesis infiltration, or post‐synthesis surface attachment.^[^ ^19^ ^]^ In the present study, the one‐pot synthesis protocol by mixing Ca^2+^, IDC ligand, and enzyme molecules in H_2_O was targeted due to its ease of operation (**Figure** **1**a). In a one‐pot synthesis protocol, the biomacromolecules are in situ encapsulated during the biocomposite crystallization via co‐precipitation^[^ ^5^ ^]^ or biomimetic mineralization mechanism.^[^ ^7^ ^]^

Herein, a biocompatible synthesis protocol for enzyme@CaIDC biocomposites was developed and optimized with the assistance of a machine learning (ML) technique. It is worth noting that we revised our developed iterative ML‐assisted training‐design‐synthesis‐measurement workflow^[^ ^20^ ^]^ by integrating the Latin Hypercube Sampling (LHS)^[^ ^21^ ^]^ algorithm during the seed data receipt selection. Compared to our previous works, this methodology modification greatly accelerates the overall ML‐assisted optimization process, identifying the best‐performing synthesis receipt for enzyme@CaIDC biocomposites within ≈ 100 experimental trials. For example, through a total of 105 trials, we identified an optimized FBSA@CaIDC (FBSA = fluorescein‐tagged bovine serum albumin) biocomposite (FBC78) with an EE value of 85.4 ± 1.7%. In addition, we demonstrated the versatility of CaIDC as an enzyme immobilization platform by successfully synthesizing FGOx@CaIDC, FCAT@CaIDC, FEST@CaIDC, and EST@CaIDC (FGOX = fluorescein‐tagged glucose oxidase, FCAT = fluorescein‐tagged catalase, FEST = fluorescein‐tagged esterase). Importantly, as compared to ZIF‐based biocomposite, enzyme@CaIDC possesses superior chemical stability in PB at various pHs, rendering it a promising class of MOF‐based biocomposite in biocatalysis applications. EST@CaIDC was chosen as a model system to showcase the potential of enzyme@CaIDC in biocatalysis. The best sample – EC19 – exhibits encapsulation efficiency (EE), retained enzymatic activity (REA), and protein loading (P_loading_) values of 28.7%, 20.1%, and 4.2 wt%, respectively.

## 2 Results and Discussion

**Figure 2:**
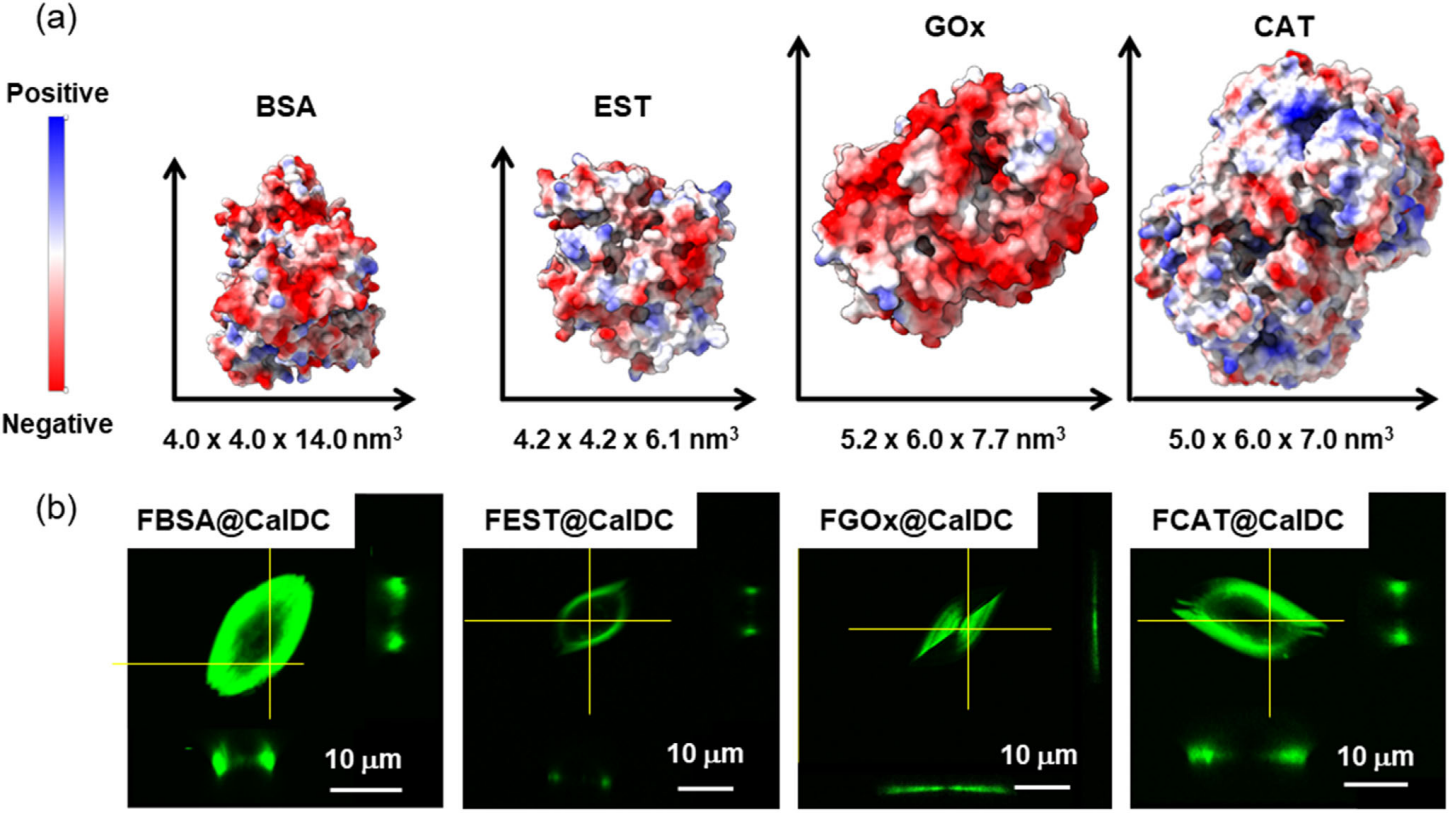
a) Molecular representations of BSA (PDB entry 4f5s^[^ ^34^ ^]^), CAT (PDB entry 1tgu^[^ ^35^ ^]^), EST (PDB entry 5fv4), and GOx (PDB entry 1cf3^[^ ^36^ ^]^) showing the coulombic surface of the enzymes. b) CLSM images of FBSA@CaIDC, FCAT@CaIDC, FEST@CaIDC, and FGOx@CaIDC.

In the literature, two Ca‐based MOFs constructed using the IDC ligand have been reported ([Ca(IDC)(H_2_O)_4_]·H_2_O and [Ca(IDC)_2_(H_2_O)_4_]·H_2_O).^[^ ^18b^ ^]^ However, their use as enzyme encapsulation platforms remains unexplored. In this context, we first attempt to examine their feasibility in a one‐pot synthesis under biocompatible synthesis conditions (neutral pH, H_2_O, room temperature). We selected FBSA as a model protein in the preliminary tests. BSA is a globular protein dimensioned at 140 × 40 × 40 Å^3^ (**Figure** **2**a),^[^ ^22^ ^]^ with an isoelectric point of 4.7.^[^ ^23^ ^]^ In the literature, BSA is commonly employed as a model protein in MOF‐based biocomposite studies prior to the use of more costly enzymes. To date, one‐pot encapsulation of BSA has been successfully demonstrated in the cases of ZIF‐8,^[^ ^7^ ^]^ UiO‐66,^[^ ^24^ ^]^ MIL‐100(Fe),^[^ ^13^ ^]^ TbBTC,^[^ ^25^ ^]^ GdBTC,^[^ ^25^ ^]^ Zn‐bdc‐NH_2_,^[^ ^26^ ^]^ etc. In the present study, the use of FBSA instead of BSA is attributed to facilitating our later attempt to use the confocal laser scanning microscopic (CLSM) technique to study the spatial localization of FBSA in/on the resulting CaIDC‐based biocomposite (vide infra).

Upon the FBSA selection, we then sought to determine the mixing order of precursors in an enzyme/CaIDC synthesis. Studies have demonstrated that the mixing order of reagents in a biocomposite synthesis can significantly impact the properties and performance of the resulting materials (e.g., EE, REA, and spatial localization of encapsulated biomolecules).^[^ ^27^ ^]^ For instance, Ge and coauthors demonstrated that adding GOx to an aqueous mixture of Zn^2^⁺ and HmIM (HmIM = 2‐methylimidazole) yields a biocomposite with the highest bioactivity, outperforming those synthesized using either a Zn^2^⁺/enzyme‐then‐ligand or a ligand/enzyme‐then‐Zn^2^⁺ approach.^[^ ^27b^ ^]^ In the present study, we adapted a synthesis protocol wherein the enzyme is first mixed with the NaOH‐deprotonated IDC ligand in H_2_O, followed by the addition of an aqueous Ca^2^⁺ solution to initiate the biocomposite formation (Figure 1a). This mixing strategy is selected based on our preliminary findings – premixing Ca^2^⁺ with EST was found to adversely affect enzyme bioactivity (Figure S1, Supporting Information). However, ^1^H NMR spectra and fluorescence spectra confirmed that neither Ca^2^⁺ norIDC ligand alone caused significant structural perturbation to EST, supporting the structural compatibility of enzymes toward the synthesis precursors (Figures S2 and S3 and S5, Supporting Information).

In the literature, a comprehensive optimization of Zn^2^⁺ and imidazolate ligand concentrations in an enzyme/ZIF synthesis to achieve enhanced encapsulation and bioactivity has been reported by our group and others.^[^ ^20^, ^28^ ^]^ Recent studies reported that an adjustment of protein concentration in a biocomposite synthesis can also influence the performance of enzyme/MOF biocomposites in terms of EE and REA.^[^ ^29^ ^]^ In our next step, we aim to explore the enzyme/CaIDC biocomposite synthesis by adjusting the concentrations of Ca^2^⁺, IDC ligand, and FBSA to potentially enhance EE value. The comprehensive investigation in this study by simultaneously optimizing the metal ion, ligand, and enzyme concentrations within a biocomposite synthesis is supported by an ML‐assisted training‐design‐synthesis‐measurement workflow developed in our previous work.^[^ ^20a^ ^]^ In the present study, the synthesis space for the CaIDC‐based biocomposite comprises 176851 different combinations of [Ca^2+^] (2–98 mm), [IDC] (22.5–122.5 mm), and [enzyme] (0.2–1.96 mg mL^−1^). Rather than selecting seed data receipts based on experience and chemical intuition as in our previous studies,^[^ ^20a^ ^]^ we applied an LHS algorithm to streamline seed data collection. Fifteen synthesis conditions from the entire synthesis space (≈0.08‰) were selected for synthesis and characterization in seed studies to serve the initialization of the ML‐assisted optimization process (Figure S6, Supporting Information). We anticipated that LHS could effectively pick representative seed conditions that broadly cover the entire synthesis search space for FBSA/CaIDC biocomposite, accelerating the overall optimization process for enzyme/CaIDC by reducing iteration cycles.

Following FBSA/CaIDC synthesis in the seed stage, we calculated the EE values for FBSA/CaIDCs by measuring the residual FBSA in the post‐synthesis supernatant via the bicinchoninic acid (BCA)^[^ ^30^ ^]^ assay (Figure S4, Supporting Information). With the synthesis receipts and EE values for the 15 seed FBSA/CaIDCs in hand (Figure S6, Supporting Information), we then trained various ML models and assessed their suitability for further study according to their mean squared error (MSE) values. Random forest (RF) was selected as it outperforms other models (gradient boosting (GB), support vector machines (SVM), and neural networks (NN)) with the lowest MSE value (Figures S7 and S8, Supporting Information). Thereafter, ten new FBSA/CaIDCs were synthesized and characterized as suggested by a Bayesian optimization (BO) algorithm based on the trained RF model, with the aim of improving EE values. The RF model was then iteratively retrained to improve its accuracy and reliability in fitting all the FBSA/CaIDC data. After nine iterative runs and a total of 105 samples (accounting for only ≈0.59‰ of the entire synthesis space for FBSA/CaIDC), we conclude the ML‐assisted optimization process upon observing convergence in the predicted maximum EE values from the RF model (Figure S10, Supporting Information). Further details on the ML model development and evaluation can be found in Supporting Information (Figures S6–S13, Supporting Information).

To determine the crystallinity of the FBSA/CaIDC samples, we carried out powder X‐ray diffraction (PXRD) measurements. Most FBSA/CaIDCs selectively crystallized as [Ca(IDC)(H_2_O)_4_]·H_2_O (termed as CaIDC, Figures 1c; S29–S34, Supporting Information) with a preferential growth on the [010] face (Figure 1c). No PXRD diffractions related to [Ca(IDC)_2_(H_2_O)_4_]·H_2_O were identified, and only three synthesis receipts yielded amorphous products (Figures S31–S34, Supporting Information). Interestingly, Ca‐IDC is reported to be synthesized in H_2_O in the presence of 10% hydrazine hydrate over several days.^[^ ^18b^ ^]^ However, our studies demonstrate that the addition of hydrazine hydrate and prolonged synthesis are not necessary for the crystallization of CaIDC. The deprotonation of the IDC ligand and its coordination to Ca^2^⁺ for the phase‐selective formation of [Ca(IDC)(H_2_O)_4_]·H_2_O can be effectively triggered by the addition of aq. NaOH at room temperature in minutes (Figures S39–S41, Supporting Information).

In the next step, we sought to summarize and analyze the EE distribution across all FBSA/CaIDCs by sorting them into pyramid (Figure 1b) and histogram (Figure S11, Supporting Information) plots. As shown in Figure 1b, among the 105 samples, the EE values for FBSA/CaIDCs ranged from 24.9% to 85.4%. FBC78 with EE and P_loading_ values of 85.4 ± 1.7% and 16.1 ± 0.1 wt.% was identified as the best FBSA/CaIDC sample (Table S1, Supporting Information). The similar EE distribution curve observed in the seed and iteration samples (Figure S11, Supporting Information) supports our hypothesis that the LHS method can effectively random‐pick seed conditions that adequately spread the entire synthesis space, reducing bias from human intuition. In our previous studies on enzyme@ZIF, we systematically selected and synthesized seed data based on experience and chemical intuition.^[^ ^20a^ ^]^ However, sample distribution analysis in our previous study revealed that this strategy failed to select synthesis receipts for seed data that generally reflect the full scope of the entire synthesis space, leaving large untapped areas that were later explored in the ML‐assisted iteration to identify enzyme@ZIFs with enhanced encapsulation and bioactivity (Figure S12, Supporting Information).^[^ ^20a^ ^]^ As a result, the optimization for GOx@ZIF or HRP@ZIF (HRP = horseradish peroxidase) required over 150 trials.^[^ ^20a^ ^]^ In contrast, in the present study, the FBC78 was identified after only 78 trials, including seed data and nine rounds of iteration. This represents a ≈ 45.8% cut in experimental workload compared to our previous studies. Thus, we propose that our revised approach of coupling LHS with ML can significantly accelerate the overall optimization protocol. Notably, this approach is expected to be adaptable beyond MOF‐based biocomposites and can be applied to other material studies incorporating ML‐driven optimization processes.

Interestingly, despite the identical crystallinity observed in most of the FBSA/CaIDC samples (Figures S30–S34, Supporting Information), varying EE values were found across different synthesis recipes (Figure 1b). A similar phenomenon was reported in enzyme@ZIFs.^[^ ^20^, ^29^ ^]^ For example, the EE values for GOx@ZIF‐8 synthesized using $\frac{\left[\mathrm{HmIM}\right]}{\left[\mathrm{Zn}\right]}$ = 4 (BZIF‐8‐B) and $\frac{\left[\mathrm{HmIM}\right]}{\left[\mathrm{Zn}\right]}$= 12 (BZIF‐8‐S) were reported to be 98.7% and 70.4%, respectively.^[^ ^26^ ^]^ Based on our literature review, we believe this phenomenon is reported for the first time in the context of carboxylate‐based MOF biocomposites.

PXRD measurement confirms that FBC78 was phase‐pure as [Ca(IDC)(H_2_O)_4_]·H_2_O (Figure 1c). CLSM was employed to examine the spatial distribution of FBSA within FBC78. As shown in **Figures** **2**b and S45c (Supporting Information), a fluorescent signal from FBSA was observed in the subsurface area of FBC78 crystals. To evaluate the possibility of surface‐adsorbed FBSA on FBC78, we also synthesized a control sample – FBSA‐on‐CaIDC – by simply mixing the as‐synthesized CaIDC crystallites with FBSA. BCA assays confirmed that a negligible amount of FBSA adsorbed onto the CaIDC surface (EE < 2%), rendering the synthesis of FBSA‐on‐CaIDC unsuccessful (Figure S24, Supporting Information). The inefficiency of surface adsorption of FBSA on CaIDC crystals may be rationalized by the hydrophilic nature of the CaIDC materials (Figure S22, Supporting Information). It is widely reported that super‐hydrophilic surfaces can act as anti‐adhesive barriers to resist protein adsorption.^[^ ^31^ ^]^ Although the surface chemistry differs from that of zwitterionic polymers,^[^ ^31^ ^]^ we hypothesize that the super‐hydrophilic CaIDC crystallites may exhibit a similar anti‐adhesive effect against protein adsorption. To further evaluate the synthesis‐performance relationship, we performed SHAP (Shapley additive explanations) analysis on the trained RF model. The SHAP analysis, presented in Figure S13 (Supporting Information), revealed that [Ca^2+^] and [IDC] are the dominant synthesis parameters influencing EE values, with higher concentrations of both leading to improved encapsulation. Considering the CLSM results and protein‐on‐CaIDC synthesis experiments, we concluded that, in FBC78, FBSA was not merely surface‐adsorbed but encapsulated within the CaIDC‐based biocomposite (FBSA@CaIDC). In addition, as no distinct differences were observed in the crystallization kinetics between the CaIDC and FBC78 samples in in situ small angle X‐ray scattering (SAXS) experiments (Figures S39 and S40, Supporting Information), we proposed that FBSA was encapsulated during the CaIDC crystallization via a co‐precipitation pathway.

To demonstrate the potential enhanced chemical stability of FBC78, we sought to examine the crystallinity and protein leaching of the sample after an 8‐h treatment in PB buffer (pH 6.0 or 7.4). PB buffer was selected as its widely use in biocatalysis applications.^[^ ^32^ ^]^ In comparison with FBC78, FBSA@ZIFs, constructed using Zn(OAc)_2_ and HmIM, were synthesized using two different $\frac{\left[\mathrm{HmIM}\right]}{\left[\mathrm{Zn}\right]}$ molar ratios ($\frac{\left[\mathrm{HmIM}\right]}{\left[\mathrm{Zn}\right]}$ = 4 (FBSA@ZIF_4_) and 12 (FBSA@ZIF_12_)). Consistent with literature reports, FBSA@ZIF_12_ crystalized as ZIF‐8, while FBSAF@ZIF_4_ as *dia*‐Zn(mIM)_2_ (Figure S43, Supporting Information).^[^ ^33^ ^]^ The EE and P_loading_ values for FBSA@ZIF_4_ and FBSA@ZIF_12_ were summarized in Table S1 (Supporting Information).

ZIF‐based biocomposites were well reported to be unstable in PB buffer.^[^ ^8^ ^]^ After PB buffer treatment (0.01 m, pH 7.4, 8 h), although all samples retained their original crystallinity as indicated by PXRD results (Figure S43, Supporting Information), ≈ 23.8 ± 0% and 4.3 ± 0% of the encapsulated FBSA were released from FBSA@ZIF_4_ and FBSA@ZIF_12_, respectively (Figure 1d). No detectable FBSA release was observed in the case of FBC78 (Figure 1d). Under a lightly acidic condition (0.01 m PB at pH 6.0, 8 h), FBSA@ZIF_4_ was dissolved with a 100% release of encapsulated FBSA within minutes, while FBSA@ZIF_12_ became amorphous with 17.4 ± 0.1% of its encapsulated FBSA released to the environment (Figures S42–S44, Supporting Information). In comparison, FBC78 retained its crystallinity during a pH 6 PB buffer treatment with 12.5% ± 0.1% FBSA release (Figures S42–S44, Supporting Information). Overall, we demonstrate that CaIDC‐based biocomposites exhibit enhanced chemical stability over ZIF‐based biocomposites, rendering them a promising platform for enzyme immobilization in biocatalysis applications.

In the next step, we seek to explore the possibility of growing core‐shell CaIDC‐based biocomposites with protein located in different layers of a CaIDC crystal. This strategy has been reported for ZIF‐based systems,^[^ ^33^ ^]^ however, it has not been reported for carboxylate‐MOF‐based biocomposites. As shown in Figure S45 (Supporting Information), by applying a layer‐by‐layer strategy, we successfully encapsulate FBC78 within FBSA@CaIDC (FBC78_2nd_). PXRD measurements confirmed that FBC78_2nd_ was phase‐pure and exhibited the same crystallinity as FBC78. SEM images confirmed the leaf‐shaped morphology of the biocomposite, which was similar to that of FBC78 but with an increased particle size (∅ = 18.4 ± 4.1 µm for FBC78 and 45.8 ± 4.9 µm for FBC78_2nd_, Figure S45 (Supporting Information). The EE and P_loading_ for FBC78_2nd_ were determined to be 48.3 ± 2.3% and 4.3 ± 0.2 wt% (Table S1, Supporting Information), respectively. Herein, we successfully demonstrated the feasibility of a layer‐by‐layer growth of CaIDC‐based biocomposites, which could serve as a useful synthesis strategy for obtaining biocomposites with multiple bioentities. Ongoing experiments are being conducted to further explore this strategy for enzymatic cascades involving multiple enzymes.

In our next step, we sought to evaluate the versatility of CaIDC as an immobilization platform for different enzymes by synthesizing FGOx@CaIDC, FCAT@CaIDC, and FEST@CaIDC using the same synthesis receipt for FBC78 but simply replacing FBSA with the respective enzymes. PXRD and SEM measurements confirmed that FGOx@CaIDC, FCAT@CaIDC, and FEST@CaIDC shared similar crystallinity and morphology as FBC78 (Figures S35 and S46, Supporting Information), while contact angle measurements revealed differences in their surface hydrophilicity (Figure S47, Supporting Information). The EE values for FGOx@CaIDC, FCAT@CaIDC, and FEST@CaIDC were measured to be 47.7 ± 5.5%, 67.8 ± 0%, and 92.5 ± 1.8%, respectively; while the P_loading_ for FGOx@CaIDC, FCAT@CaIDC, and FEST@CaIDC were determined to be 0.3 ± 0.03 wt.%,0.2 ± 0 wt.%, and 0.7 ± 0.01 wt.%, respectively (Table S1, Supporting Information). The synthesis yield (Y in %) of the biocomposites (excluding enzyme, calculated based on IDC) was 85.8%, 71.6%, and 76.8% for FGOx@CaIDC, FCAT@CaIDC, and FEST@CaIDC, respectively, and 66.0% for FBC78. These results indicates a good synthetic efficiency under mild aqueous conditions (Table S1, Supporting Information). The spatial distributions of enzymes within FGOx@CaIDC, FCAT@CaIDC, and FEST@CaIDC were examined using CLSM (Figure 2b). Interestingly, the spatial distribution of FGOx in FGOx@CaIDC was found to differ from other biocomposites. Rather than being encapsulated in the subsurface of the CaIDC crystal edge, FGOx was observed to be encapsulated in the plane of the leaf‐like CaIDC crystals. We suspected that the differences in encapsulation patterns and immobilization parameters for different CaIDC‐based biocomposites were attributed to the distinct surface chemical properties of the proteins. It is well‐documented that the surface chemistry of a protein can influence the success of biocomposite synthesis,^[^ ^37^ ^]^ the growth pattern of the biocomposite,^[^ ^38^ ^]^ the protein distribution within the composite,^[^ ^7^, ^33^, ^38^ ^]^ and its immobilization parameter (EE, P_loading_, and REA).^[^ ^39^ ^]^ The isoelectric points for BSA, CAT, EST, and GOx were reported to be 4.7,^[^ ^23^ ^]^ 5.4,^[^ ^40^ ^]^ 5.0,^[^ ^41^ ^]^ and 4.2,^[^ ^42^ ^]^ respectively. GOx was reported to be more negatively charged on the surface as compared to other studied enzymes. To further investigate this protein‐induced structure‐performance difference, we sought to examine the zeta potential (ζ) of the fluorescein‐tagged proteins. As expected, the studied enzymes possess different surface‐charged in aq. PB solution (pH = 7.0) – ζ_FGOx_ = −18.18 mV, ζ_FEST_ = −10.21 mV, ζ_FBSA_ = −7.26 mV, and ζ_FCAT_ = −6.22 mV (Figure S48, Supporting Information). Notably, FGOx possesses a significantly more negative charge (−18.18 mV) as compared to the other proteins (FBSA, FEST, and FCAT), which follows the trend of reported isoelectric points of the enzymes studied. Based on these results, we hypothesize that the highly negatively charged GOx may preferentially coordinate to the exposed Ca^2^⁺ sites on the (010) facet of CaIDC crystallites, while other enzymes with less negative surface charges are more likely to associate with the (100) facets where the imidazole ligands are more exposed (Figure S29, Supporting Information). Nonetheless, as only four enzymes were examined in this study, a more systematic investigation with a broader range of biomacromolecules possessing diverse zeta potentials will be pursued in future work to further substantiate this hypothesis.

**Figure 3:**
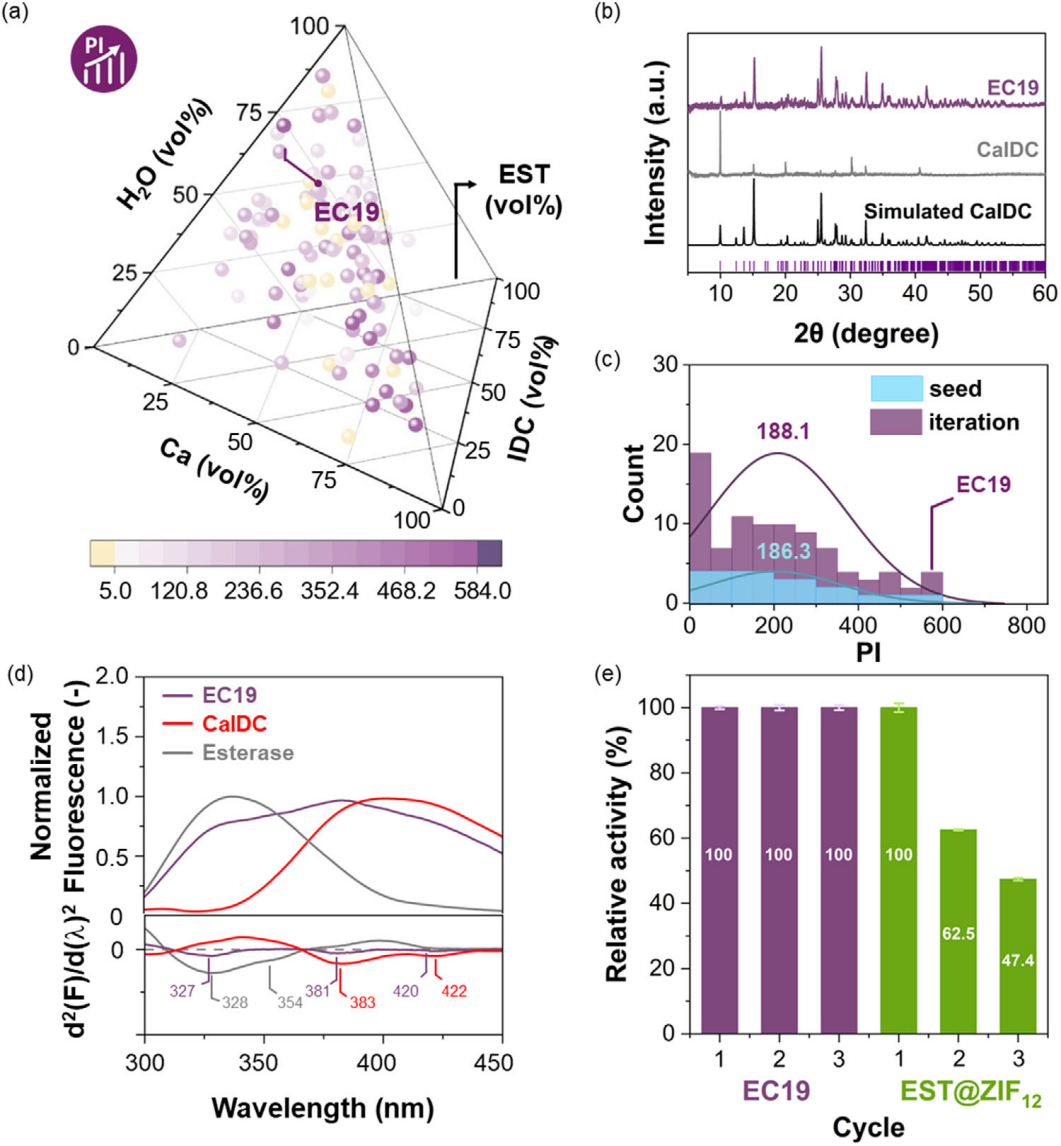
a) 3D pyramid plot showing the PI values for EST@CaIDCs in both the seed and the iteration stages. The color scale and spot size indicate the degree of PI value. b) PXRD patterns of EC19 (purple) and CaIDC (grey). c) Histograms showing the distributions of EST@CaIDC according to their PI values. Samples from the seed data set and iteration stage are shown in blue and purple, respectively. d) Fluorescence spectra and second derivative fluorescence spectra (λ_ex_ = 280 nm) of esterase (grey), CaIDC (red), and EC19 (purple). The fluorescence spectra shown in the main text focus on the 300–450 nm range, while the full spectra are provided in Figure S27 (Supporting Information). e) Cycling runs for the hydrolysis of NPA using EC19 and EST@ZIF_12_.

Last, to demonstrate the applicability of enzyme@CaIDC in biocatalysis, we sought to optimize the synthesis of the EST@CaIDC. The synthesis, optimization, and characterization protocols were analogous to those for FBSA@CaIDCs (vide supra), with the use of a performance index (performance index (PI) = EE × REA) in the ML‐assisted optimization workflow instead of using only EE. Detailed information on the ML model development can be found in the Supporting Information (Figures S15–S21, Supporting Information). Hydrolysis of *p*‐nitrophenyl acetate (NPA, Figure S14, Supporting Information) was employed as a model assay reaction to determine the REA value of the biocomposites. To ensure that the observed catalytic activity originates solely from the enzyme encapsulated in CaIDC, we performed control experiments on the individual components of the system. As shown in Figure S1 (Supporting Information), while both Ca^2^⁺ and the IDC ligand exhibit slight catalytic activity in the hydrolysis reaction, their reaction kinetics are at least an order of magnitude slower than those observed in the presence of esterase. This confirms that the catalytic performance of esterase@CaIDC is primarily attributed to the encapsulated enzyme. The REA and EE values for all 105 synthesized samples across the seed (15 samples) and iteration stages (90 samples in 9 iterations) are summarized and presented in Figure S20 (Supporting Information). EC19 was identified as the best EST@CaIDC biocomposite (**Figure** **3**a,c), achieving EE = 28.7 ± 3.8%, REA = 20.1 ± 4.8%, and P_loading_ = 4.2 ± 0. wt% (Table S2, Supporting Information). The success of EST encapsulation in EC19 was confirmed by transmission electron microscopic studies (Figure S25, Supporting Information).

It is noteworthy to mention that the pH of the synthesis mixture of EC19 after mixing all the ingredients was measured to be pH 8.89, which corresponds to a mildly alkaline yet biocompatible condition. As compared to the enzyme@ZIF, we proposed that the synthesis condition for Ca‐IDC‐based biocomposite is more biocompatible (pH > 14 for enzyme@ZIF).^[^ ^33^ ^]^ In an enzyme@ZIF synthesis, an excess amount of imidazole ligand was generally required to facilitate ligand deprotonation and Zn‐ligand coordination.^[^ ^33^ ^]^ To further evaluate the pH evolution during the synthesis process, we monitored the pH at multiple time points using two representative samples, FBC78 and EC19. The initial aqueous NaOH‐deprotonated IDC ligand solutions exhibited strong alkalinity, with pH values of 10.58 and 10.99, respectively. Upon mixing with the enzyme and Ca^2+^, the pH dropped rapidly within 5 min to 7.96 for FBC78 and 8.89 for EC19, respectively. The pH values of the synthesis mixture remained relatively stable over time, reaching 7.86 for FBC78 and 8.48 for EC19, respectively, at 20 h. These results suggest that, despite the initial alkalinity of the ligand solutions, the reaction environment quickly equilibrates to a mildly alkaline condition that is more biocompatible to the enzyme molecules.

Interestingly, the PXRD pattern of EC19 revealed the presence of multiple crystal faces even before mechanical grinding, in contrast to the FBC78 sample which exhibited a strong [010] preferential orientation prior to grinding (Figures 1c and 3b, and Figure S38, Supporting Information). This observation suggests that the interaction between EST and CaIDC during crystallization impacted on the directional growth of the crystals, leading to a more isotropic morphology with multiple crystal faces naturally exposed. To further corroborate this observation, SEM imaging was performed to visulize the morphological differences between the two samples. As shown in Figures S44 and S23 (Supporting Information), while FBC78 displayed a single, well‐defined leaf‐like structure, EC19 exhibited a multi‐layered morphology with multiple leaf‐like sheets stacked together. These distinct morphologies indicate that the presence of EST in the EC19 synthesis condition may interfere with the preferential growth of CaIDC along the [010] plane, thereby promoting the formation of a more isotropic crystal structure with multiple exposed facets.

To evaluate the porosity of EC19, we employed non‐linear density functional theory (NLDFT) pore size distribution analysis based on 77K N_2_ sorption isotherms. The results revealed mesoporosity within the bio‐composite (Figure S28, Supporting Information). This mesoporosity may provide additional diffusion channels for substrates and products during the hydrolysis reaction, which helps explain the observed REA of the samples.

Although CaIDC possesses mesopores in the range of ≈4.5–6.8  nm, the pristine framework shows limited overall porosity and low surface area (S_BET_ = 14.5  m^2^  g^−1^ for EC19, Figure S28, Supporting Information). In contrast, ZIF‐8 – a widely used MOF in enzyme immobilization – exhibits a significantly higher surface area (≈1462.8  m^2^  g^−1^) and micropores in the range of 0.3–1.2  nm. In CaIDC, its limited porosity likely contributes to the lower catalytic efficiency observed (Table S2, Supporting Information).

To understand the tertiary structure of EST in EC19, we measured the ATR‐FTIR (ATR‐FTIR = Attenuated total reflectance Fourier transform infrared spectroscopy) spectrum of the biocomposite and compared the results with free EST (Figure S26, Supporting Information). Due to the low EST loading in EC19, the peak at ≈1672 cm^−1^ originated from the C═O stretching of the coordinative IDC ligand in CaIDC (Figure S26, Supporting Information) strongly overlaps with the amide I signals of EST appearing at ≈1640 cm^−1^ in the IR spectrum of EC19, preventing a detailed IR analysis to understand the tertiary structure of encapsulated enzymes. We then switched to analyze and compare the fluorescence emission spectra of EC19 and free EST. For EST, which contains multiple tryptophan residues (PDB 5fv4: Trp‐57, −121, −158, −172, −183, −289, −364, −481, −498, −528), the fluorescence emission spectrum reflects a composite signal arising from these different residues. To better resolve and compare the individual contributions, we applied a second‐order derivative analysis to the fluorescence emission spectra of EST, CaIDC, and EST@CaIDC samples. As shown in Figures 3d and S27 (Supporting Information), two main peaks were identified in the fluorescence emission spectrum of free EST (λ_max_ = 328 and 354 nm). As compared to free EST, no distinct differences in fluorescence maxima were observed for encapsulated EST in EC19, implying an undisturbed tertiary structure of EST in EC19. These findings are further supported by the fluorescence spectra of EST in the presence of IDC ligand, as shown in Figure S5 (Supporting Information), where no significant change was observed in fluorescence signal in terms of the peak position. However, the presence of Ca(OAc)_2_ was found to partially distort the EST tertiary structure (Figure S5, Supporting Information). These effects of synthesis precursors (Ca) fluorescence spectroscopic results also indicate that the precursors themselves do not disrupt the tertiary structure of the enzymes prior to encapsulation.

To further understand the underlying synthesis‐performance relationship, we performed a t‐distributed stochastic neighbor embedding (t‐SNE) analysis (Figure S21, Supporting Information) to visualize the distribution of EE and REA values across the synthesis space. The results revealed that optimal synthesis regions for a high EE and high REA occupy distinct areas. Since our ML optimization targeted an enhanced PI in the resulting EST/CaIDC biocomposite, it effectively identified synthesis conditions that balance both parameters. This finding explains why the optimized sample EC19 exhibits moderate individual EE and REA values rather than extreme values for either parameter.

Ca‐MOMs were reported to be another class of versatile platform for enzyme immobilization applications.^[^ ^12^ ^]^ To compare the performance between EST@CaBDC and EC19, we synthesized and characterized EST@CaBDC following the reported protocol.^[^ ^4h^ ^]^ As shown in Table S2 (Supporting Information), EC19 (EE = 28.7%, P_loading_ = 4.2 wt.%, and REA = 20.1%) exhibited superior immobilization performance over EST@CaBDC (EE = 43.8 ± 1.14%, P_loading_ = 0.94 ± 0.02 wt.%, and REA = 8.1 ± 3.2%). The difference EST immobilization parameters observed between EC19 and EST@CaBDC samples likely due to the differences in the chemical nature of CaIDC versus CaBDC and/or the lack of synthesis optimization for EST@CaBDC. However, the relatively low EE and REA values observed for EC19, compared to some literature‐reported biocomposites,^[^ ^43^ ^]^ can be attributed to the synthesis strategy, which was designed to simultaneously optimize both parameters rather than to maximize either one individually, as supported by the t‐SNE analysis. Additionally, the relatively low enzyme loading (P_loading_ = 4.2 wt.%) in EC19 may have contributed to its moderate overall catalytic performance. These limitations may also stem from the hydrophilic nature and/or the low porosity of the CaIDC platform (Figures S22 and S28, Supporting Information). To further improve the catalytic performance in CaIDC‐based biocomposite, we propose that a potential future research direction will be to rationally enhance the hydrophobicity via ligand engineering,^[^ ^43^, ^44^ ^]^ to increase the porosity of the CaIDC platform via defect engineering,^[^ ^45^ ^]^ and/or to introduce dopant additives^[^ ^9^, ^46^ ^]^ in the enzyme@CaIDC synthesis to optimize enzyme‐MOF biointerface.

Lastly, we demonstrated that EC19 can be successfully recycled at least three times with negligible bioactivity loss (Figure 3e). PXRD analysis confirmed that EC19 retained its crystallinity throughout the recycling process (Figure S36, Supporting Information). In comparison, EST@ZIF_12_ lost 52.6% of its original bioactivity after three cycles (Figure 3e), which we attributed to enzyme leaching during the reaction due to the poor structural stability of the ZIF‐based biocomposite (Figure S37, Supporting Information). After a 24‐hour treatment in PB buffer (0.01 m, pH 7.4), 100% and 22.3% of the encapsulated esterase leached out from EST@ZIF_4_ and EST@ZIF_12_, respectively; whereas EC19 exhibited minimal enzyme leaching (<2%).

## 3 Conclusion

In conclusion, we have developed a biocompatible synthesis protocol for a CaIDC‐based biocomposite in water at room temperature. Compared to ZIF‐based systems, CaIDC‐based biocomposites exhibited superior chemical stability in PB buffer (pH 6.0 and 7.4), making them a promising platform for enzyme immobilization in biocatalysis applications. Various enzymes (FBSA, FGOx, FCAT, FEST, and EST) were effectively encapsulated in CaIDC via a one‐pot coprecipitation process. By employing an ML‐assisted workflow to fine‐tune the concentrations of Ca^2+^, IDC, and enzyme in the synthesis, optimal synthesis formulations for biocomposites with enhanced encapsulation and bioactivity were efficiently identified. Importantly, integrating LHS into the ML‐assisted workflow significantly reduced experimental trials while maintaining robust optimization outcomes. In an EST‐based model system, the optimized biocomposite (EC19) achieved notable performance metrics with EE of 28.7%, REA of 20.1%, and P_loading_ of 4.2 wt%. This study highlights the potential of CaIDC as a versatile and chemically stable platform for enzyme encapsulation in biocatalysis. Furthermore, the LHS‐coupled BO optimization strategy, akin to artificial natural selection, provides a powerful and efficient toolbox for advancing biomolecule protection and enzyme immobilization technologies.

## 4 Experimental Section

All chemicals and solvents were purchased from commercial sources and used as received without further purification. MilliQ water was used in all experiments.

### Fluorescein‐Tagged Enzymes

Fluorescein isothiocyanate (FITC, 0.5 mg) and enzyme (40 mg BSA (bovine serum albumin, Sigma Aldrich)), EST (esterase from the porcine liver, Sigma Aldrich), GOx (glucose oxidase from *Aspergillus niger*, Sigma–Aldrich), CAT (catalase from bovine liver, Sigma–Aldrich)) were dissolved in a carbonate‐bicarbonate aqueous buffer solution (0.1 m, pH 9.2, 4 mL) and left overnight at 4 °C. The FITC‐tagged enzymes (FITC‐tagged enzymes: FBSA, FEST, FGOx, FCAT) was recovered by passing the reaction mixture through an Illustra NAP‐25 column (GE Healthcare Life Sciences, NSW, Australia). The crude enzyme solution was concentrated through a 10K membrane by centrifugation (4000 rpm for 10 min), followed by solvent exchange with ultrapure water. The concentration‐solvent‐exchange process was repeated two times to ensure the buffer salts were completely removed from the solution. The obtained fluorescein‐tagged enzyme solution was stored in water in darkness at 4 °C.

### Systematic Screening of FBSA/CaIDC (or EST/CaIDC)

In a typical set of seed data for FBSA/CaIDC, a desired volume of aqueous stock solution of Ca(OAC)_2_·H_2_O (Reagent grade, Ajax Finechem Pt., Ltd), Imidazole‐4,5‐dicarboxylic acid (IDC, Merck), FBSA, and H_2_O were mixed. The concentration of the enzyme stock solution was 2 mg mL^−1^. In the present study, to a synthesis protocol was adhered wherein H_2_O, IDC stock solution (100 mm, deprotonated using 1 N NaOH), and FBSA stock solution were first combined and vortexed for 1 min. Subsequently, 100 mm Ca(OAc)_2_·H_2_O solution was added to the mixture. The final synthesis was conducted under rotation at room temperature for 20 h to ensure consistency. The volume of the synthesis is 0.5 mL. After synthesis, the precipitates were collected by centrifugation (148 000 rpm for 2.5 min) and washed three times with MilliQ water. The collected FBSA@CaIDC was resuspended in MilliQ water (0.5 mL). The collected supernatant was subjected to BCA assay (BCA Protein Assay Kit, for 20–2000 µg mL^−1^ protein, Sigma–Aldrich) for encapsulation efficiency (EE in %). The EST/CaIDC has the same procedure for systematic screening, only replacing FBSA with EST. The collected EST/CaIDC was subjected to activity assay for retained enzymatic bioactivity (REA in %) determination.

### Synthesis of FBC78 and CaIDC

In a typical synthesis of FBC78, 122 µL of H_2_O was mixed with 73 µL of IDC stock solution (125 mm) in a 2 mL centrifuge tube. Thereafter, 50 µL of FBSA (2 mg mL^−1^) was introduced and vortexed for 1 min. After the addition of 254 µL of Ca(OAc)_2_ stock solution (100 mm), the mixture was rotated at room temperature for 20 h. The washing procedure for FBC78 was identical to other FBSA/CaIDC samples in the systematic study. The CaIDC has the same synthesis procedure for FBC78, only replacing FBSA with H_2_O.

### Synthesis of EC19

In a typical synthesis of EC19, 357 µL of H_2_O was mixed with 10 µL of IDC stock solution (125 mm) in a 2 mL centrifuge tube. Thereafter, 123 µL of EST (2 mg mL^−1^) was introduced and vortexed for 1 min. After the addition of 357 µL of Ca(OAc)_2_ stock solution (100 mm), the mixture was rotated at room temperature for 20 h. The washing procedure for EC19 was identical to other EST/CaIDC samples in the systematic study.

### Synthesis of ZIF_4_ and FBSA@ZIF_4_

In a typical synthesis of FBSA@ZIF_4_, 100 µL of H_2_O was mixed with 200 µL of HmIM (98%, Sigma–Aldrich) stock solution (400 mM) in a 2 mL centrifuge tube. Thereafter, 100 µL of FBSA (2 mg mL^−1^) was introduced and vortexed for 1 min. After the addition of 200 µL of Zn(OAc)_2_·2H_2_O (Reagent grade, Chem‐Supply) stock solution (100 mm), the mixture was rotated at room temperature for 20 h. The synthesized FBSA@ZIF_4_ was washed three times with MilliQ water. The ZIF_4_ has the same synthesis procedure for FBSA@ZIF_4_, only replacing FBSA with H_2_O.

### Synthesis of ZIF_12_ and FBSA@ZIF_12_

In a typical synthesis of FBSA@ZIF_12_, 100 µL of H_2_O was mixed with 200 µL of HmIM (98%, Sigma–Aldrich) stock solution (1600 mm) in a 2 mL centrifuge tube. Thereafter, 100 µL of FBSA (2 mg mL^−1^) was introduced, and vortexed for 1 min. After the addition of 200 µL of Zn(OAc)_2_·2H_2_O (Reagent grade, Chem‐Supply) stock solution (100 mm), the mixture was rotated at room temperature for 20 h. The synthesized FBSA@ZIF_12_ was washed three times with MilliQ water. The ZIF_12_ has the same synthesis procedure for FBSA@ZIF_12_, only replacing FBSA with H_2_O.

### Powder X‐Ray Diffraction (PXRD)

PXRD measurements were performed on a Rigaku SmartLAB SE powder diffractometer using Cu Kα (λ = 1.5406 Å) radiation.

### Attenuated Total Reflection Fourier Transform Infrared Spectroscopy (ATR‐FTIR) Analysis

ATR‐FTIR Measurements were carried out on a Thermo Nicolet iS50 infrared spectrometer. Repeated spectra were collected in the spectral range from 2200 to 400 cm^−1^, averaging 128 scans at 4 cm^−1^ spectral resolution. ATR‐FTIR spectra were collected and analyzed using the OMNIC software.

### Confocal Laser Scanning Microscopy (CLSM)

The presence and spatial location of the fluorophore‐tagged biomolecules in (or on) the MOF composites were determined using CLSM technique (Olympus FV3000 confocal laser scanning microscope, Olympus). The fluorescein‐tagged esterase was excited at 488 nm and the fluorescence signal was collected in a window from 495 to 545 nm. Although image deconvolution techniques can enhance clarity,^[^ ^47^ ^]^ the native CLSM images in this study already sufficiently revealed enzyme distribution within the MOF; thus, deconvolution was not applied.

### Scanning Electron Microscopy (SEM)

SEM images were collected using a Zeiss Sigma HD. Prior to analysis, the samples were dry‐loaded or dispersed in H_2_O by sonication, drop‐cast on a 12 mm aluminum SEM stage, and sputter‐coated with a 5 mm platinum thin film.

### In Situ Small Angle X‐Ray Diffraction (SAXRD)

In situ SAXRD experiments were conducted on an Anton‐Paar SAXS point diffractometer. In a typical experiment, the synthesis solution of CaIDC or enzyme/CaIDC was prepared in a 25 mL glass tube with a total volume increased to 5 mL (10 times the original volume) while maintaining the same component ratios. The solution was continuously injected into a quartz capillary (∅ = 1 mm) placed in the X‐ray beam using a peristaltic pump, ensuring constant stirring during the injection process. SAXRD measurements commenced 1 min after the preparation of the synthesis solution. SAXRD patterns of CaIDC or enzyme/CaIDC samples were recorded at 10 s intervals over a duration of 5 min.

### 77K N_2_ Sorption Analysis

Gas adsorption isotherms were measured on an Autosorb IQ physisorption analyzer. Approximately 20 mg of the FBC78 or EC19 was placed into a glass analysis tube and degassed under a dynamic vacuum for 6 h at 220 °C prior to measurement. Nitrogen (N_2_) adsorption and desorption isotherms were measured at 77K. The isotherms were then analyzed to determine the BET surface area using the ASiQwin software. Pore size distribution was modeled from the N_2_ adsorption isotherms using the non‐linear density functional theory (NLDFT) model.

### Zeta‐Potential Measurements

100 µL of FBSA, FEST, FGOx, or FCAT (2 mg mL^−1^) was added to 900 µL of 0.01 m pH 7.0 PB buffer. The zeta potentials of the protein samples were measured using a Malvern ZetaSizer dynamic light scattering instrument with a folded capillary zeta‐potential cell.

### Solution‐State Nuclear Magnetic Resonance (NMR)

All solution‐state NMR experiments were conducted on a Bruker Avance III 400 MHz NMR spectrometer. All NMR data were analyzed using Bruker Topspin v4.2.0.

### Quantification of EE in Enzyme/CaIDC

To quantitatively measure the encapsulation efficiency (EE) of enzymes in enzyme/CaIDC, a BCA assay was conducted on the supernatant of enzyme/CaIDC samples. The BCA working reagent was prepared by mixing 16 mL of Reagent A (Bicinchoninic acid solution, Sigma‐Aldrich) with 1 mL of Reagent B (Copper(II) sulfate solution, 4% (w v^−1^), Sigma–Aldrich). The enzyme solution (2 mg mL^−1^) was serially diluted to concentrations of 0.125, 0.25, 0.5, and 1 mg mL^−1^, and 40 µL of each diluted solution was mixed with 800 µL of BCA working reagent. The mixtures were incubated in a preheated oven (60 °C) for 15 min and then analyzed for absorbance at 562 nm using a UV–vis spectrometer (SPECTROstar Nano, BMG LABTECH). A standard calibration curve was prepared using the free enzymes to determine enzyme concentrations (Figure S4, Supporting Information). For the enzyme/CaIDC samples, 40 µL of supernatant was mixed with 800 µL of BCA working reagent and heated at 60 °C for 15 min. After incubation, residual pellets were removed via centrifugation at 14800 rpm, and the concentration of unencapsulated enzymes in the supernatant was calculated based on the standard curve and absorbance at 562 nm. Protein assays were performed in triplicate, and the EE (%) for each enzyme/CaIDC sample was calculated using the following equation:

(1) $$\begin{array}{c} \mathrm{Enca}\mathrm{psul}\mathrm{atio}\mathrm{neff}\mathrm{icie}\mathrm{ncy}\left(\mathrm{EE}\right)=\\ \frac{\left(m_{\mathrm{enzy}\mathrm{meus}\mathrm{edin}\mathrm{thes}\mathrm{ynth}\mathrm{esis}}-m_{\mathrm{enzy}\mathrm{mein}\mathrm{thes}\mathrm{ynth}\mathrm{esis}\mathrm{supe}\mathrm{rnat}\mathrm{ant}}\right)}{m_{\mathrm{enzy}\mathrm{meus}\mathrm{edin}\mathrm{thes}\mathrm{ynth}\mathrm{esis}}}\% \end{array}$$

### Enzymatic Assay for EST@CaIDC

The activity of EST/CaIDC was assessed by measuring the rate of p‐nitrophenol release during the hydrolysis of p‐NPA. In a typical assay, solution A was prepared by mixing 7.842 mL of PBS buffer (0.01 m, pH 7.4) with 158 µL of 50 mm p‐nitrophenyl acetate (p‐NPA) dissolved in dimethyl sulfoxide. In a 96‐well plate, 30 µL of EST (0.01 mg mL^−1^) or EST/CaIDC suspension (0.01 mg mL^−1^, based on EST) was added to 200 µL of solution A. The absorbance of the reaction mixture at 348 nm was immediately monitored using a SPECTROstar Nano spectrometer. The initial reaction rate was used to quantify the enzymatic bioactivity of the free enzyme and enzyme/CaIDC composites. The retained enzymatic bioactivity (REA, in %) for each enzyme/CaIDC composite was calculated using the following equation:

(2) $$\mathrm{Reta}\mathrm{ined}\mathrm{enzy}\mathrm{mati}\mathrm{cbio}\mathrm{acti}\mathrm{vity}\left(\mathrm{REA}\right)=\frac{A_{\mathrm{enzy}\mathrm{me}/\mathrm{CaIDC}}}{A_{\mathrm{free}\mathrm{enzy}\mathrm{me}}}\%$$

### Reusability of EC19

The reusability of EC19 was examined. After a 5‐min reaction, the EC19 was collected by centrifugation while the supernatant was preserved for UV quantification. The recovered EC19 was washed three times with H_2_O to remove any residual substrate in the system. Thereafter, EC19 was reintroduced into a fresh reaction medium and conditioned, and it was left to react for another reaction.

### Machine Learning Algorithm

In this study, five machine learning algorithms were tested: gradient boosting (GB), support vector machines (SVM), neural networks (NN), random forests (RF), and Gaussian process regression (GP). For each algorithm, two separate regression models were developed using the seed dataset, with the aim of predicting EE and REA.

A 10‐fold cross‐validation strategy was employed to assess model performance. This approach is commonly used to evaluate a model's ability to generalize to unseen data, providing a more reliable and consistent performance estimate compared to a single train/test split. In this work, the chosen performance metric is the mean square error (MSE).

Among the five tested machine learning algorithms, random forest (RF) demonstrated the highest accuracy and was selected as the most suitable model for this study.

### ML‐Assisted Experiment Planning

The Bayesian optimization (BO) algorithm was employed in conjunction with a random forest (RF) model to identify promising FBSA/CaIDC (or EST/CaIDC) candidates. During the iteration stage, FBSA/CaIDC (or EST/CaIDC) designs were selected to maximize the expected improvement (EI) acquisition function, which is defined as:

(3) $$f\left(\vec{x}\right)=Z\sigma \left(\vec{x}\right)\phi \left(Z\right)+\sigma \left(\vec{x}\right)\phi \left(Z\right)$$

(4) $$Z=\left\{\frac{\left(\mu \left(\vec{x}\right)-f^{\prime }\xi \right)}{\begin{array}{c} \sigma \left(\vec{x}\right)\\ 0,\sigma \left(\vec{x}\right)=0 \end{array}}\sigma \left(\vec{x}\right)\right.>0$$

where $\mu \left(\vec{x}\right)$ is the predicted mean EE/PI (PI = EE x REA) from the RF, *f*′ is the current largest mean EE/PI predicted by the model, $\sigma \left(\vec{x}\right)$ is the standard deviation of EE/PI from the RF, Φ, and ∅ are the cumulative and probability density functions of the normal distribution, respectively. ξ (ξ = 0.01 in the present study) is a hyperparameter that controls the balance between exploring untapped regions of the chemical space and exploiting known regions of it to achieve FBSA/CaIDC (or EST/CaIDC) with high EE/PI.

Specifically, 30 FBSA/CaIDC (or EST/CaIDC) designs were generated using the BO algorithm and ranked in descending order based on their predicted EE or PI values. From these, the top 10 candidates were selected iteratively for further evaluation.

The sampling strategy during the exploitation stage followed a similar approach to that used in the iteration stage, except that the probability improvement (PI) function was employed in the BO algorithm instead of the expected improvement function.

(5) $$f\left(\vec{x}\right)=\phi \left(\frac{f\left(\vec{x}\right)-f^{\prime }-\xi }{\sigma \left(\vec{x}\right)}\right)$$

Where

Φ is the cumulative distribution function of the standard normal distribution

ξ is a parameter that controls the trade‐off between exploration and exploitation (ξ = 0.01 in the present study)

*f*′ is the best EE/PI predicted by the model

$\sigma (\vec{x})$ is the predicted standard deviation at x

## Conflict of Interest

The authors declare no conflict of interest.

## Supporting information



Supporting Information

## Data Availability

The data that support the findings of this study are available in the supplementary material of this article.
