# Supplementary material for: One‐Pot Encapsulation of Enzymes in a Calcium Carboxylate Metal‐Organic Framework for Improved Buffer Stability
Source: Adv Sci (Weinh). 2025 Oct 2;12(44):e10960. doi: 10.1002/advs.202510960 (PMC12667492; doi:10.1002/advs.202510960)
Supplement: Supplementary file 1 — Supporting Information [file ADVS-12-e10960-s001.docx]

Supporting Information for

**One-Pot Encapsulation of Enzymes in a Calcium Carboxylate Metal-Organic Framework for Improved Buffer Stability**

Ying Shu,^a^ Weibin Liang,^a^* Jun Huang^a^*

^a^School of Chemical and Biomolecular Engineering, University of Sydney, Darlington, NSW 2008, Australia

**Table of Content**

| Content | Page |
| --- | --- |
| S1 Control experiments | S2 |
| S2 Machine learning model development and optimization results | S3 |
| S3 Characterization of FBC78 and EC19 | S15 |
| S4 Crystal structure analysis | S18 |
| S5 Formation kinetics of CaIDC and enzyme@CaIDC | S23 |
| S6 Stability evaluation of FBC78 compared to FBSA@ZIF_4_ and FBSA@ZIF_12_ | S25 |
| S7 Layer-by-Layer growth of FBC78 | S27 |
| S8 Versatility of CaIDC as an enzyme immobilization platform | S28 |
| S9 Immobilization parameter summary for samples | S28 |
| S10 References | S30 |

**S1 Control experiments**


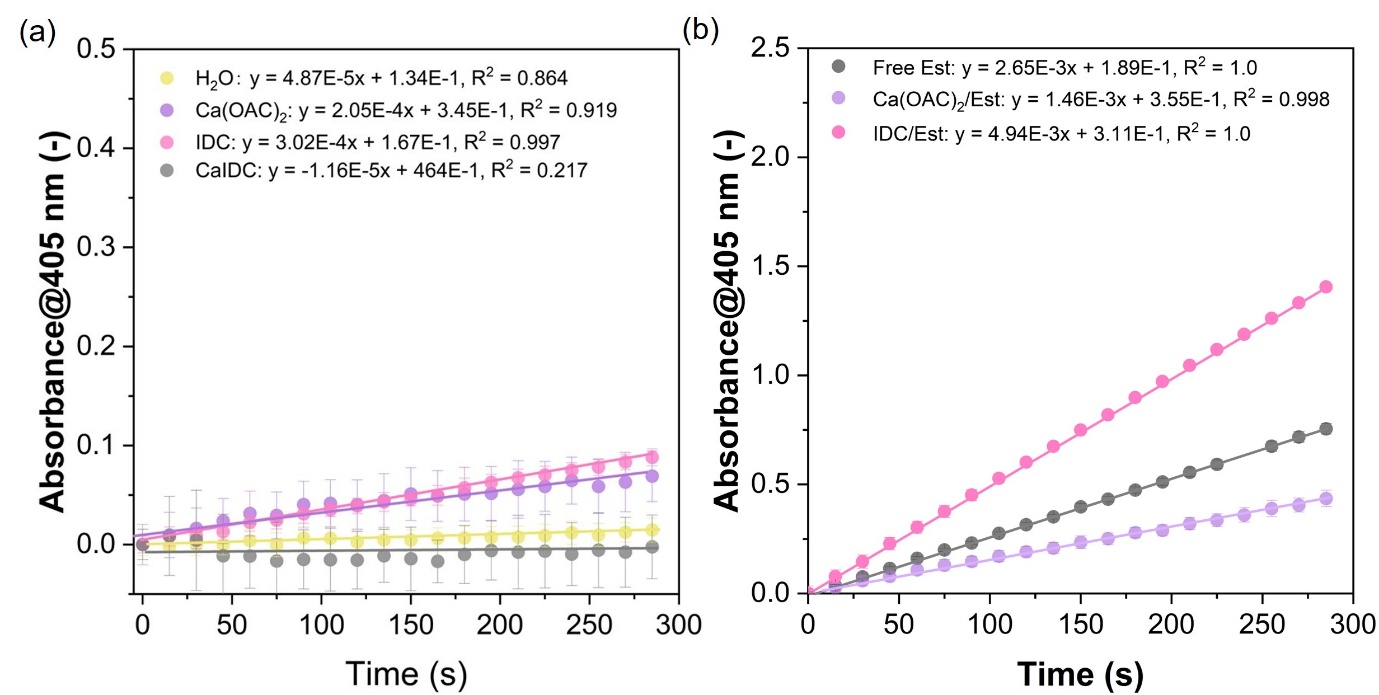


Figure S1 (a) Catalytic activity of H_2_O (yellow), Ca(OAc)_2_ (purple), IDC (pink), and CaIDC (grey) for the Ester Hydrolysis Reaction. (d) Influence of the Ca(OAc)_2_ (purple) and IDC (pink) on esterase activity, with a reference line showing the catalytic activity of free esterase (grey).

To investigate the potential influence of MOF precursors (Ca²⁺ and IDC ligand) and CaIDC MOF on esterase bioactivity, we conducted a series of control experiments. As shown in Figure S1a, CaIDC does not catalyze the p-nitrophenyl acetate hydrolysis reaction, whereas Ca²⁺ and H₃IDC exhibit slight catalytic activity. Figure S1b demonstrates that Ca²⁺ suppresses esterase bioactivity, while IDC enhances it.


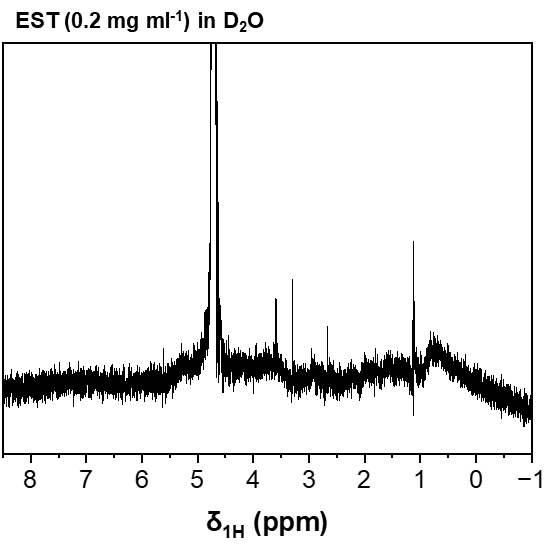


Figure S2 ^1^H NMR spectrum of EST (0.2 mg ml^-1^ in D_2_O).


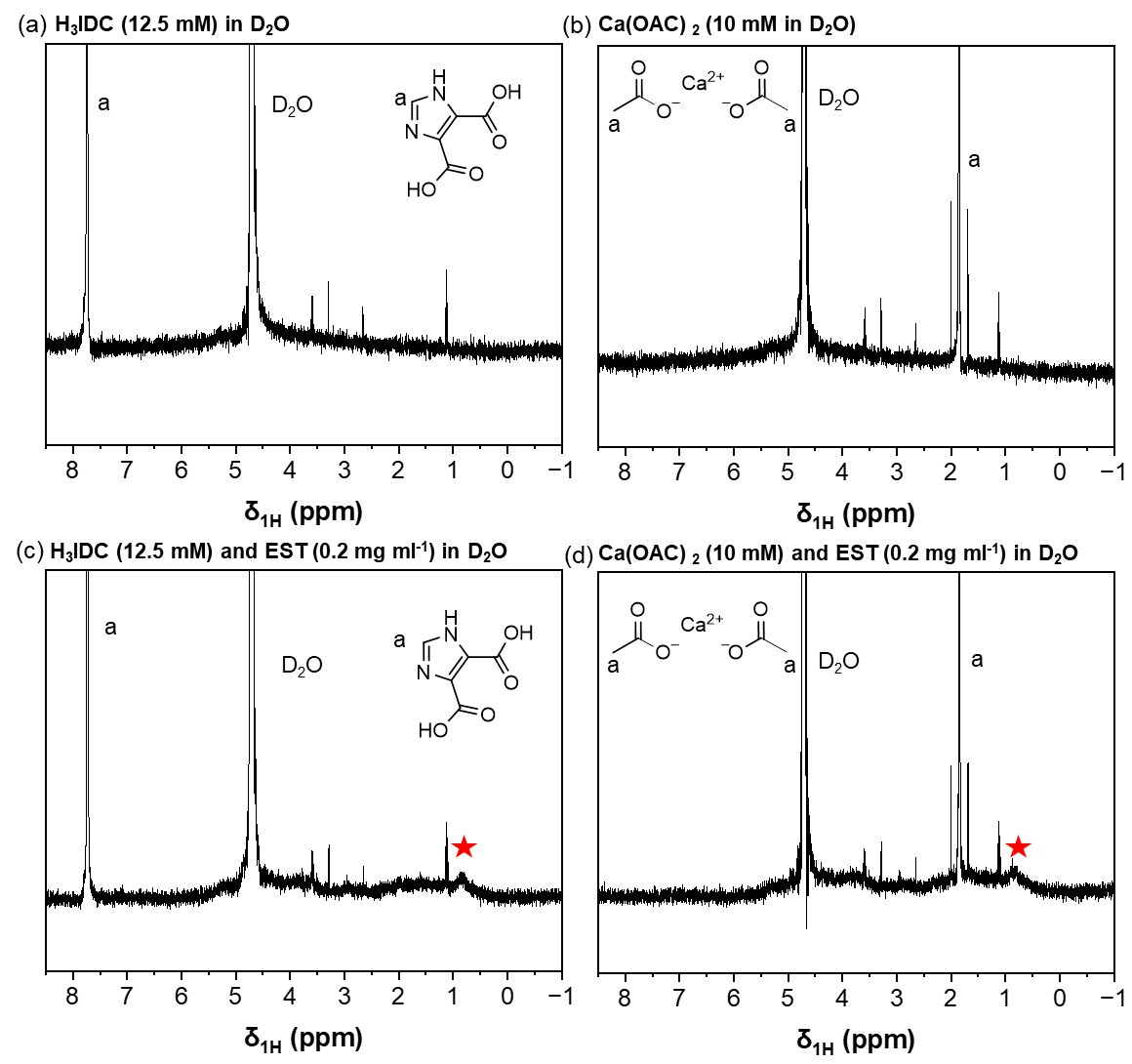


Figure S3 ^1^H NMR spectra of (a) IDC (12.5 mM in D_2_O), (b) Ca(OAC)_2_ (10 mM in D_2_O), (c) IDC (12.5 mM) and EST (0.2 mg ml^-1^) in D_2_O, and (Ca(OAC)_2_ (10 mM) and EST (0.2 mg ml^-1^) in D_2_O.

The presence of distinct signals in the high-field region (0–1 ppm), typically attributed to shielded methyl groups from hydrophobic residues such as leucine, isoleucine, and valine (Figure S2), indicates that the esterase adopts a folded conformation.^[1]^ Notably, these high-field peaks remain clearly visible upon the addition of either IDC or Ca(OAc)₂, suggesting that the protein maintains its structural integrity and does not undergo significant unfolding in either system.


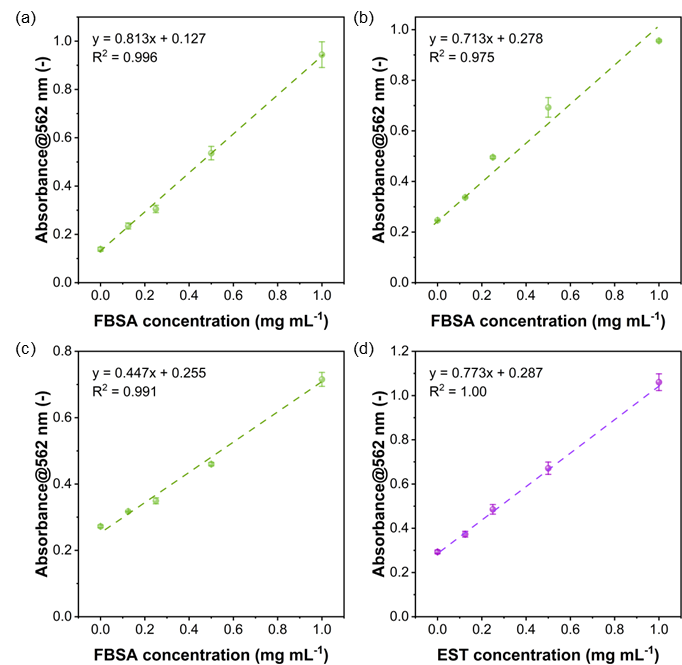


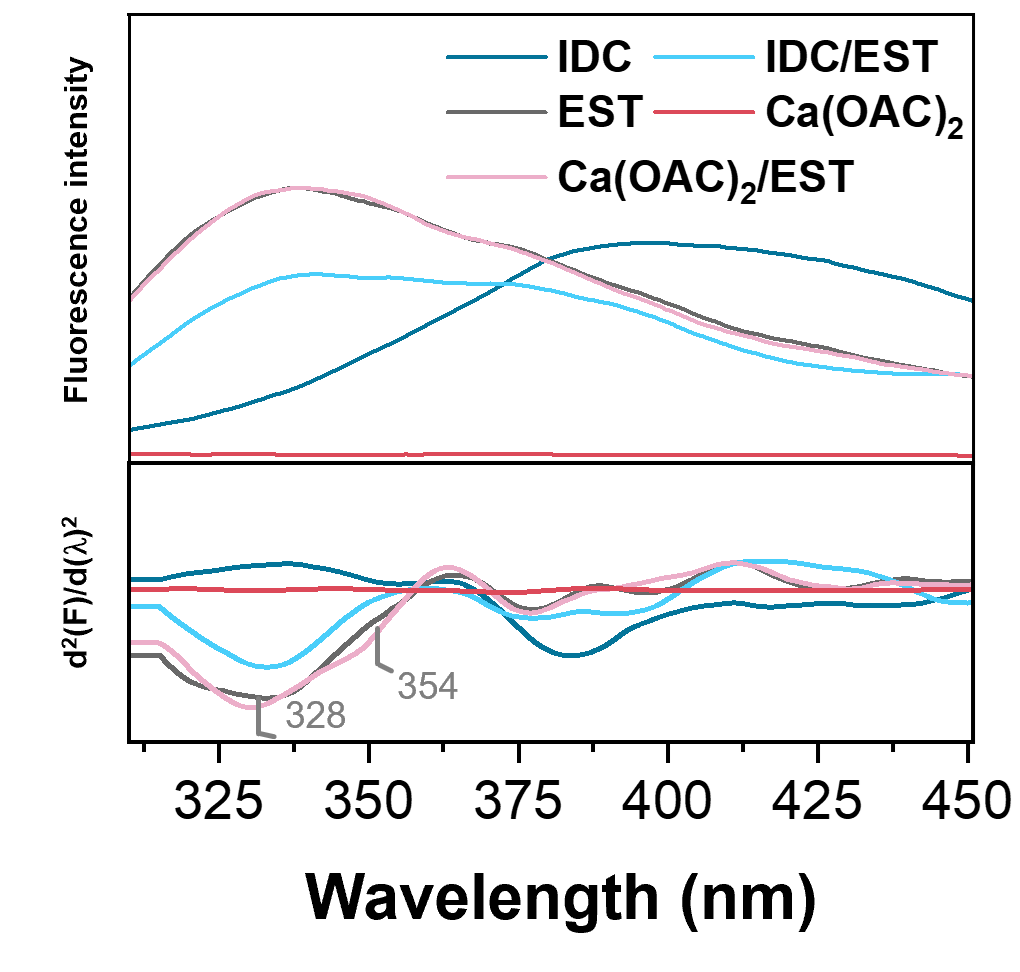
Figure S4 The BCA calibration curves for FBSA in H₂O (a), FBSA in pH 6.0 PB buffer (b), FBSA in pH 7.4 PB buffer (c), and EST in H₂O (d). Data points represent the mean values from triplicate measurements.

Figure S5 Influence of the Ca(OAc)_2_ and IDC on EST fluorescence spectra. Excitation wavelength: 280 nm. Experimental conditions: 50  μL of 2  mg mL^-1^ EST solution was mixed with 50  μL of 100  mM Ca(OAc)₂ or IDC solution, and the total volume was adjusted to 500  μL with ultrapure water prior to measurement.

To evaluate the structural influence of synthesis precursors on enzymes, fluorescence emission spectra of EST was recorded in the presence of Ca(OAc)₂ and IDC. Fluorescence spectroscopy is highly sensitive to the microenvironment of Trp residues and is widely used to monitor protein conformational changes.^[2]^ EST contains multiple Trp residues (PDB 5fv4, Trp-57, -121, -158, -172, -183, -289, -364, -481, -498, -528), and its native emission spectrum shows two major peaks at 328 nm and 354 nm. Upon addition of Ca(OAc)₂, these peaks slightly blue-shifted to 326 nm and 349 nm, respectively. According to literature, such blue shifts suggest that Trp residues are relocated to a less polar, more hydrophobic environment, likely due to reduced exposure to polar water molecules.^[2-3]^ In contrast, IDC exhibits an intrinsic fluorescence peak at ~385 nm but did not affect the Trp fluorescence of EST, suggesting negligible perturbation of its tertiary structure. These fluorescence spectroscopic results corroborate well with the enzymatic results shown in Figure S5, which demonstrates that the presence of IDC ligand has a negligible effect on the enzymatic activity of EST; however, the presence of Ca(OAc)_2_ decreases the EST enzymatic activity.

**S2 Machine learning model development**

**S2.1 ML-assisted encapsulation optimization for FBSA/CaIDC**


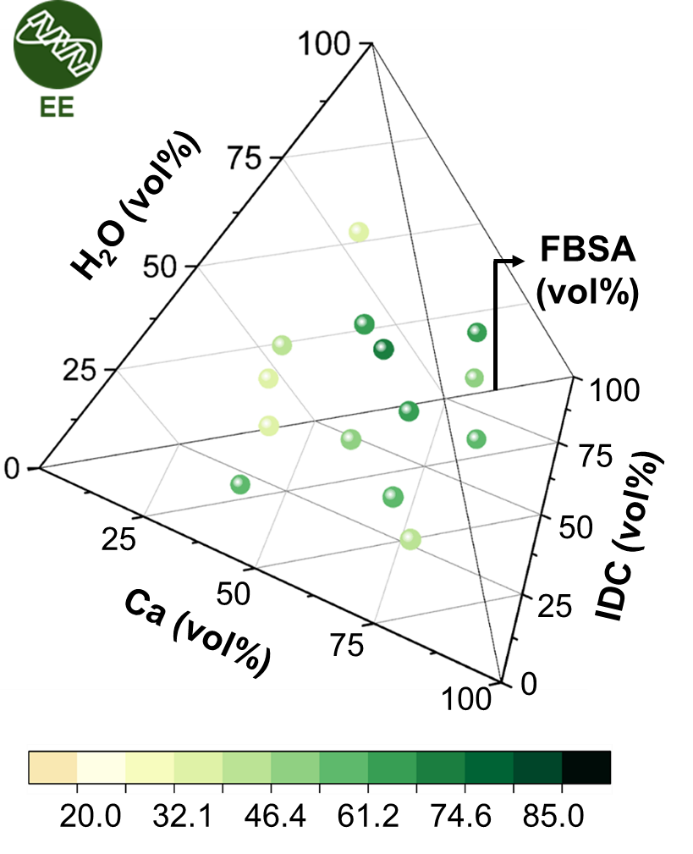


Figure S6 Pyramid plot of all 15 distinct synthesis recipes for FBSA@CaIDC in the seed study. The color scale and spot size represent the degree of EE values (%).


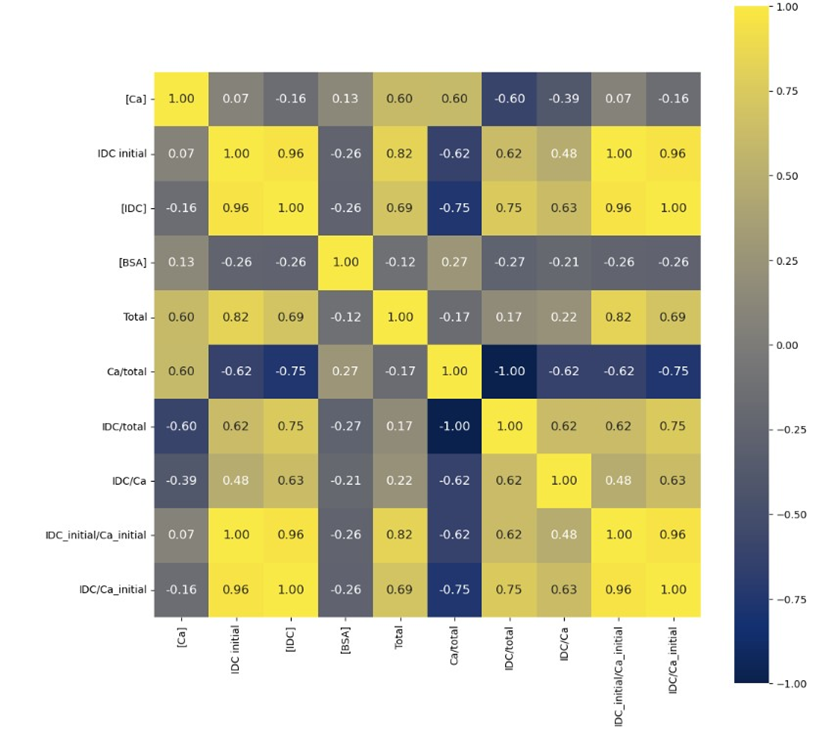


Figure S7 Heat map illustrating the correlation between synthesis features in the FBSA/CaIDC syntheses. Synthesis features include: (1) molar concentration of aqueous Ca^2+^ prior to its addition to an aqueous mixture of HIDC and FBSA ([Ca_ini_]; (2) molar concentration of Ca^2+^ in the final synthesis medium of FBSA/CaIDC ([Ca]); (3) molar concentration of IDC in the IDC and FBSA aqueous mixture prior to the addition of Ca^2+^ ([IDC_ini_]); (4) molar concentration of IDC in the final synthesis medium of FBSA/CaIDC ([IDC]); (5) total molar concentration of MOF components in the FBSA/CaIDC synthesis medium ([Ca]+[IDC]); (6) fraction of [Ca] to the total molar concentration of MOF components ( [Ca]/([Ca]+[IDC]) ); (7) fraction of [IDC] to the total molar concentration of MOF components ([IDC]/([Ca]+[IDC])); (8) IDC-to-Ca molar ratio ([IDC]/[Ca] ); (9) IDC_ini_-to-Ca_ini_ molar ratio ([IDC_ini_]/[Ca_ini_] ); and (10) IDC-to-Ca_ini_ molar ratio ([IDC]/[Ca_ini_]).


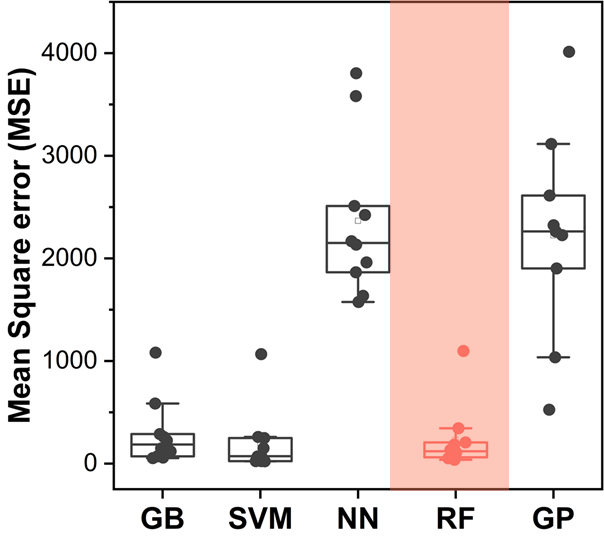


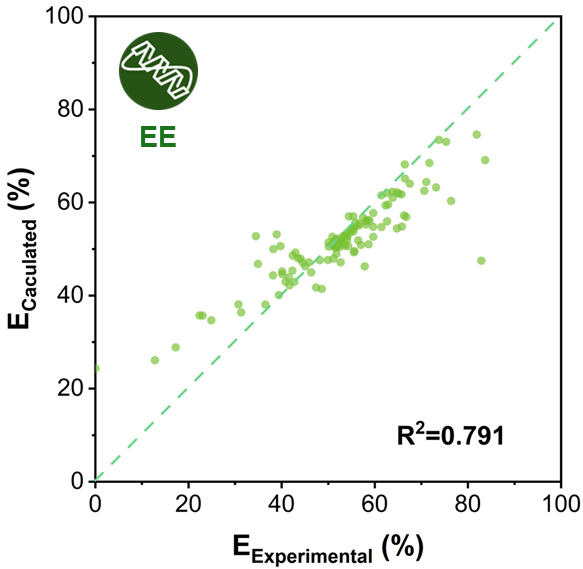
Figure S8 (a) Boxplots showing the mean square error (MSE) values from K = 10 cross-validations for models based on gradient boosting (GB), support vector machine (SVM), neural network (NN), and random forest (RF). The models were trained to predict EE values. Seed data shown in Figure S6 was used for model training.

Figure S9 Correlational plots showing the relationship between experimentally measured EE values and the RF model predicted values. The accuracy of the RF model in fitting the experimental data is emphasized by the corresponding R^2^ values, listed as inset in the Figure.


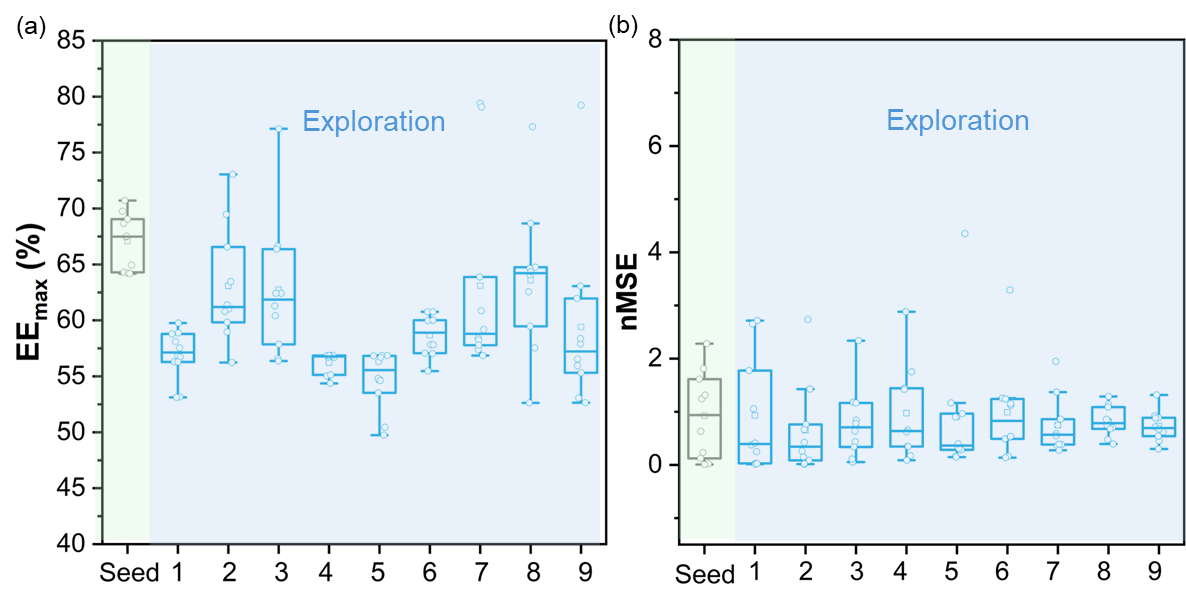


Figure S10 (a). Box plots showing the evolution of the EE values among nine FBSA/CaIDC suggestions according to the BO algorithm (x 10 suggestions) as a function of iteration number. (b) Box plots showing the evolution of the normalized mean square error (nMSE) for EE values among nine FBSA/CaIDC suggestions.


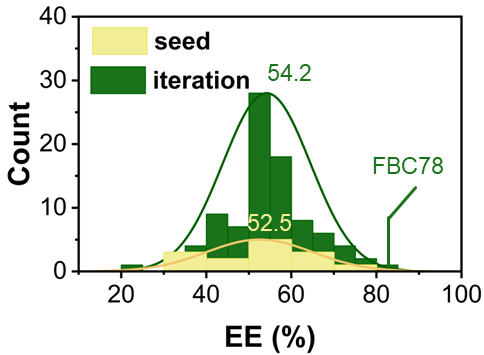


Figure S11 Histograms showing the distributions of FBSA/CaIDCs according to their EE values. Samples from the seed data set and iteration stage are shown in yellow and green, respectively.


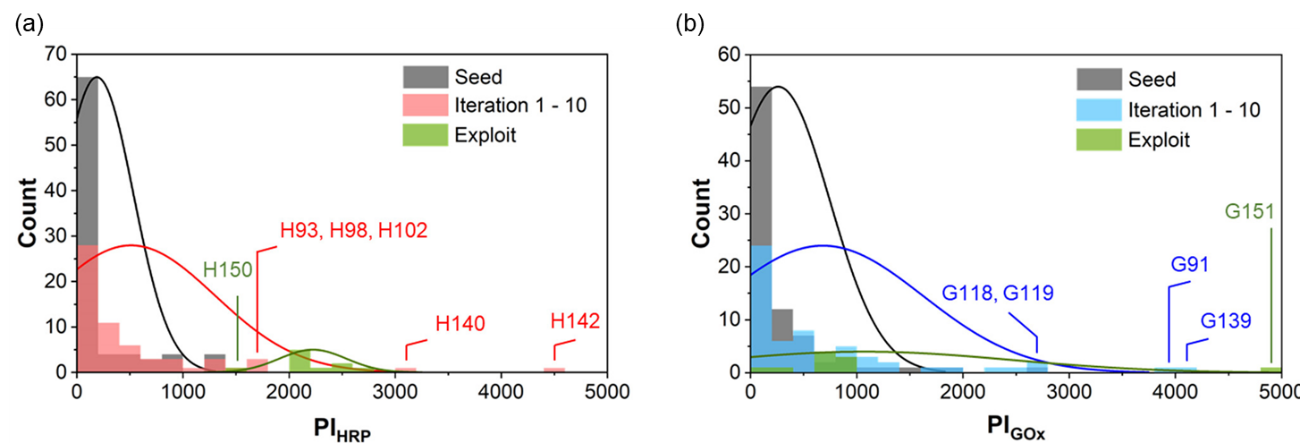


Figure S12 (a, b) Histograms showing the distributions of HRP/ZIFs (a) and GOx/ZIFs (b) according to their PI values. Samples from the seed data set and exploitation stage are shown in grey and green, respectively. Reproduced with permission.^[4]^ Copyright 2023, American Chemical Society.


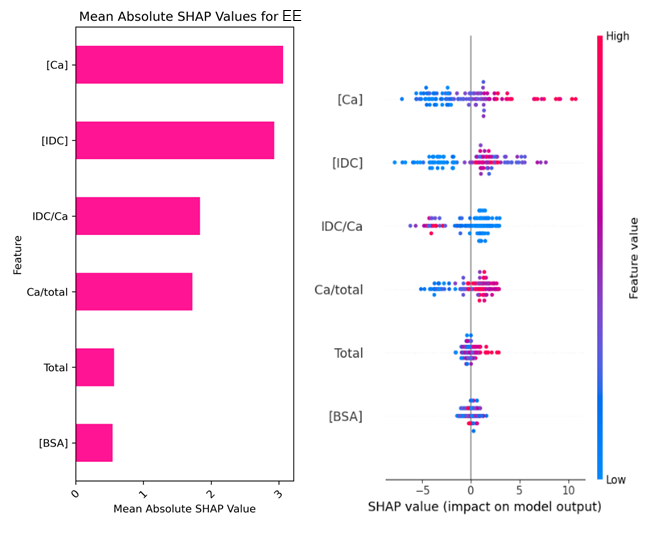


Figure S13 Mean Absolute Shapley additive explanations (SHAP) values (left) and SHAP value (right) for FBSA/CaIDC to quantify the importance of synthesis features on the prediction of the EE values.

To understand the synthesis-performance relationship in the FBSA@CaIDC biocomposite, we performed SHAP (Shapley additive explanations) analysis on the trained RF model. Although SHAP analysis is based on the synthesis feature analysis in a trained RF model, the trained RF model accurately predicts the EE values of FBSA/CaIDC (R² = 0.791, Figure S9). The accuracy of our trained RF model demonstrates the feasibility of using SHAP analysis to reflect the synthesis-performance relationship in the FBSA@CaIDC biocomposite.

As shown in Figure S13, SHAP analysis reveals that [Ca^2+^] and [IDC] are the two most important synthesis features in predicting EE values, with higher concentrations of [Ca^2+^] and/or [IDC] leading to higher EE values. Interestingly, [BSA] did not have a significant impact on the resulting EE values for the biocomposite.

**S2.2 EST@CaIDC: Performance prediction and optimization**


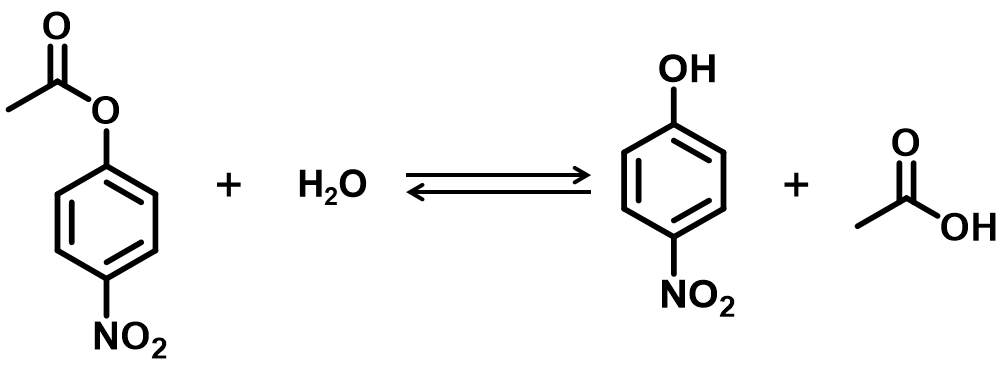


Figure S14 Hydrolysis reaction of p-NPA to para-nitrophenol (p-NP).


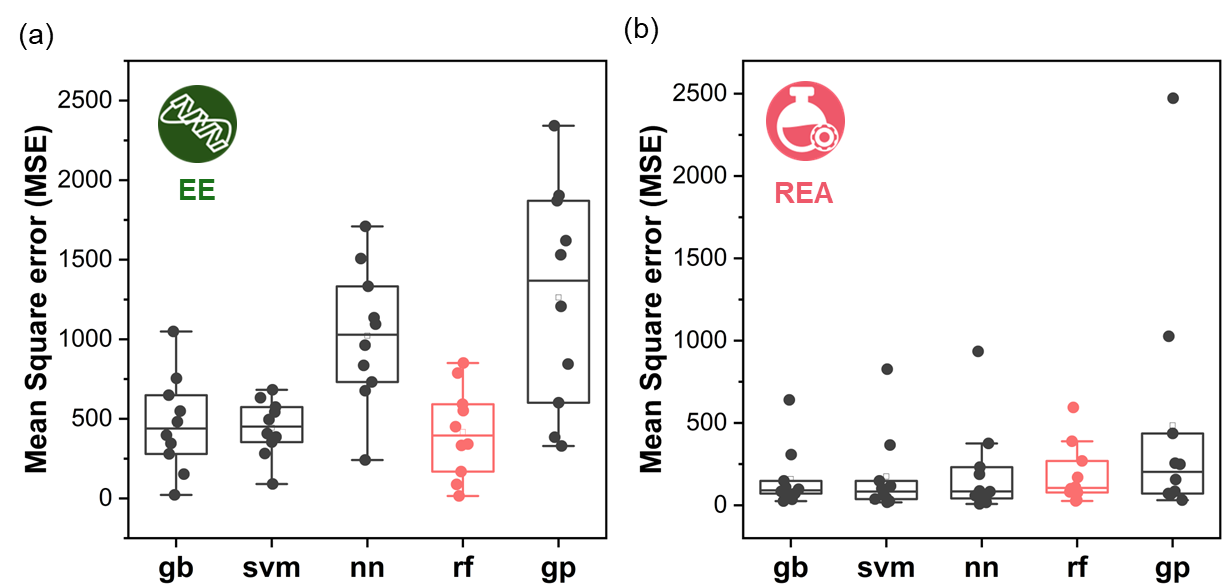


Figure S15 Boxplots showing the mean square error (MSE) values from K = 10 cross-validations for models based on GB, SVM, NN, and RF. The models were trained to predict EE (a) and REA (b) values.


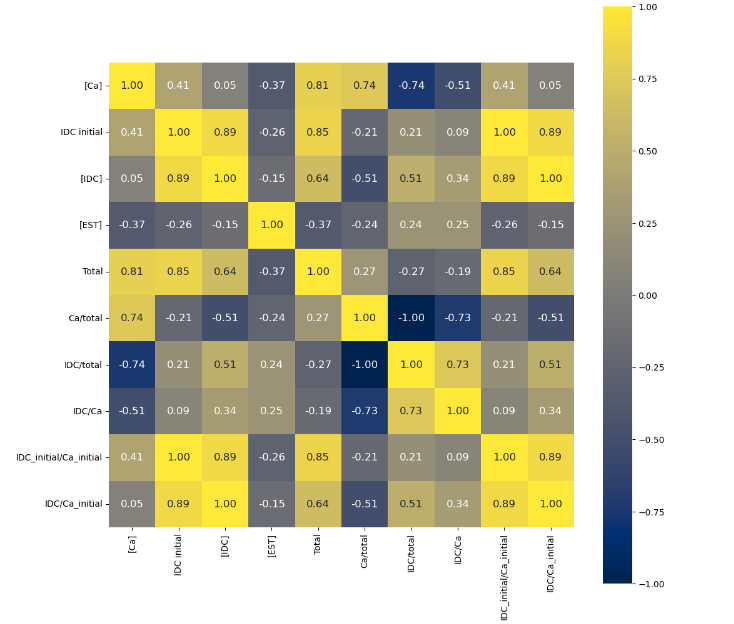


Figure S16 Heat map illustrating the correlation between synthesis features in the EST/CaIDC syntheses. Synthesis features include: (1) molar concentration of aqueous Ca^2+^ prior to its addition to an aqueous mixture of HIDC and EST ([Ca_ini_]; (2) molar concentration of Ca^2+^ in the final synthesis medium of EST/CaIDC ([Ca]); (3) molar concentration of IDC in the IDC and EST aqueous mixture prior to the addition of Ca^2+^ ([IDC_ini_]); (4) molar concentration of IDC in the final synthesis medium of EST/CaIDC ([IDC]); (5) total molar concentration of MOF components in the EST/CaIDC synthesis medium ([Ca]+[IDC]); (6) fraction of [Ca] to the total molar concentration of MOF components ( [Ca]/([Ca]+[IDC]) ); (7) fraction of [IDC] to the total molar concentration of MOF components ([IDC]/([Ca]+[IDC])); (8) IDC-to-Ca molar ratio ([IDC]/[Ca] ); (9) IDC_ini_-to-Ca_ini_ molar ratio ([IDC_ini_]/[Ca_ini_] ); and (10) IDC-to-Ca_ini_ molar ratio ([IDC]/[Ca_ini_]).

The correlation heatmap (Figure S16) provides a comprehensive visualization of the relationships between features in the dataset, highlighting key patterns of multicollinearity and independence. Features like [EST] exhibit relatively weak correlations with most other variables, indicating their independence and potential as unique predictors in the model.


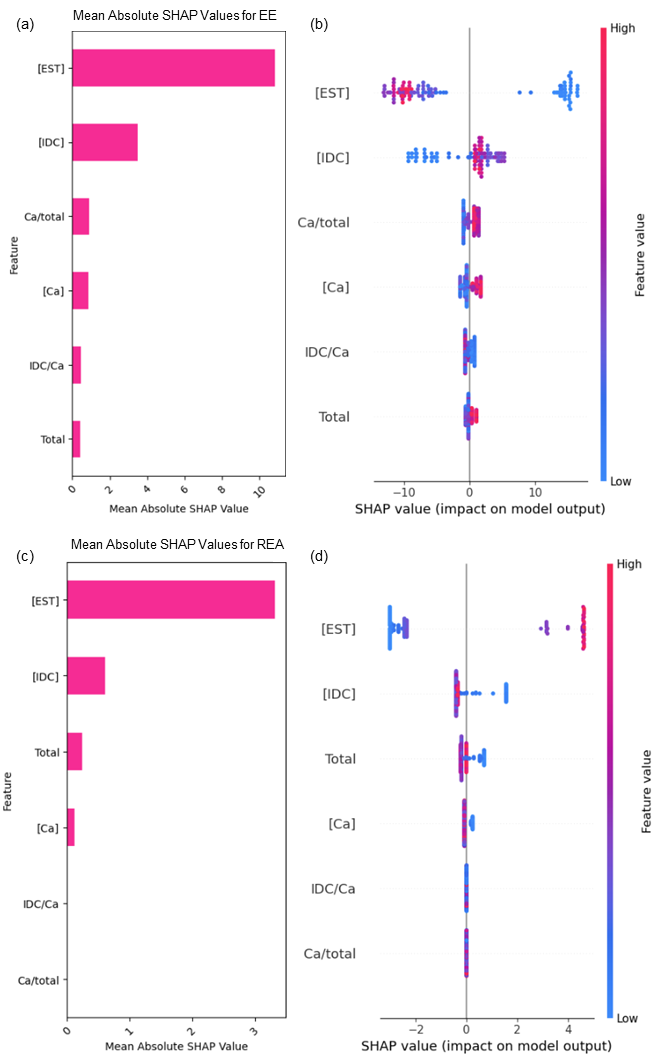


Figure S17 (a) Mean Absolute SHAP values and (b) SHAP values for EST/CaIDC to quantify the importance of synthesis features on the prediction of the EE values. (b) Mean Absolute SHAP values and (c) SHAP values for EST/CaIDC to quantify the importance of synthesis features on the prediction of the REA.

The SHAP value analysis in Figure S16 reveals the primary factors influencing the MOF system's enzyme EE and REA values. [EST] emerges as the most significant feature, demonstrating the highest mean absolute SHAP values and the widest distribution, highlighting its critical role in enhancing both EE and REA values. [IDC], while secondary, also contributes meaningfully, likely reflecting its influence on the structural or chemical environment affecting enzyme activity. Conversely, features such as [Ca], IDC/Ca, and Ca/total show negligible impact, indicating their limited importance in the current model. These findings emphasize the central importance of [EST] and its optimization in improving MOF-based enzyme delivery systems, with secondary consideration given to [IDC] for further enhancement of system performance.


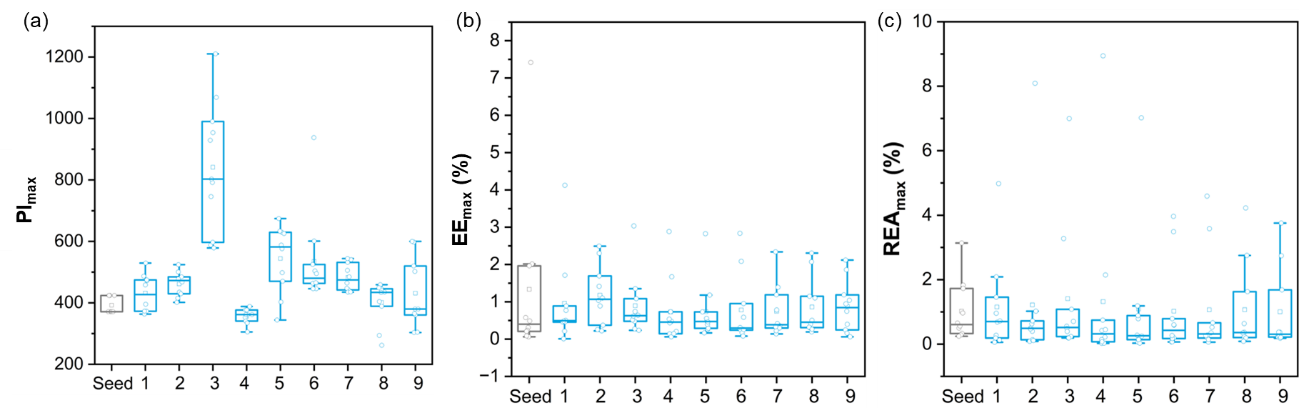


Figure S18 (a) Box plots showing the evolution of PI values among nine EST/CaIDC suggestions according to the BO algorithm (x 10 suggestions) as a function of iteration number. Box plots showing the evolution of the nMSE for (b) EE and (c) REA values among nine EST/CaIDC suggestions.


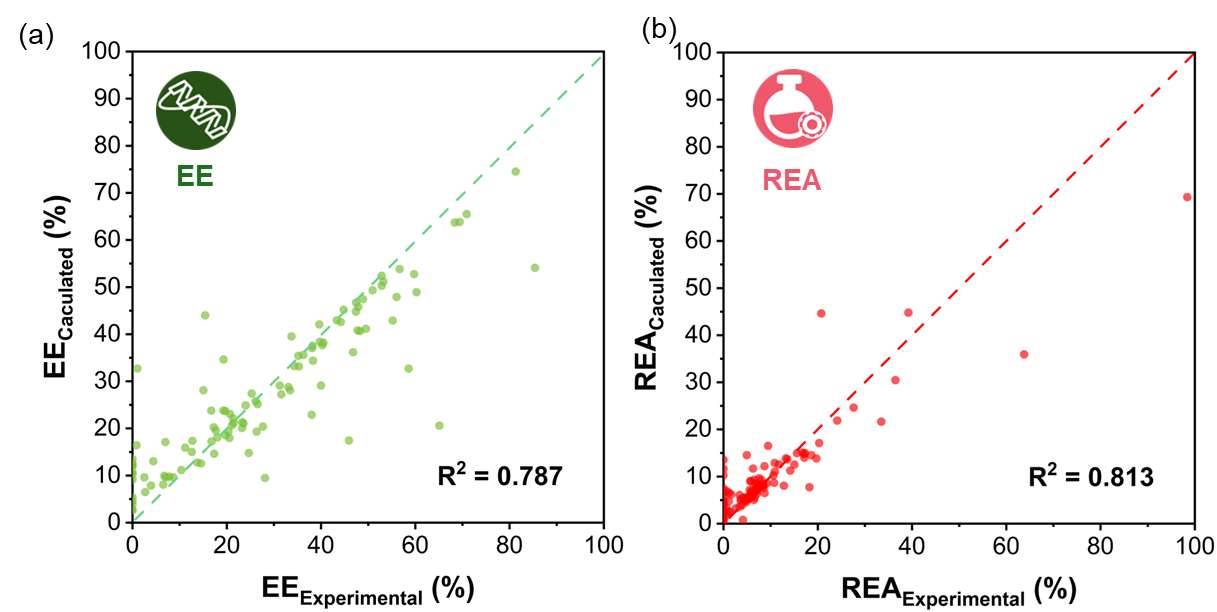


Figure S19 Correlational plots showing the relationship between experimentally measured EE (EE in %, green), REA (REA in %, red) values, and the RF model predicted values. The accuracy of the RF model in fitting the experimental data is emphasized by the corresponding R^2^ values, listed as insets in the Figures.


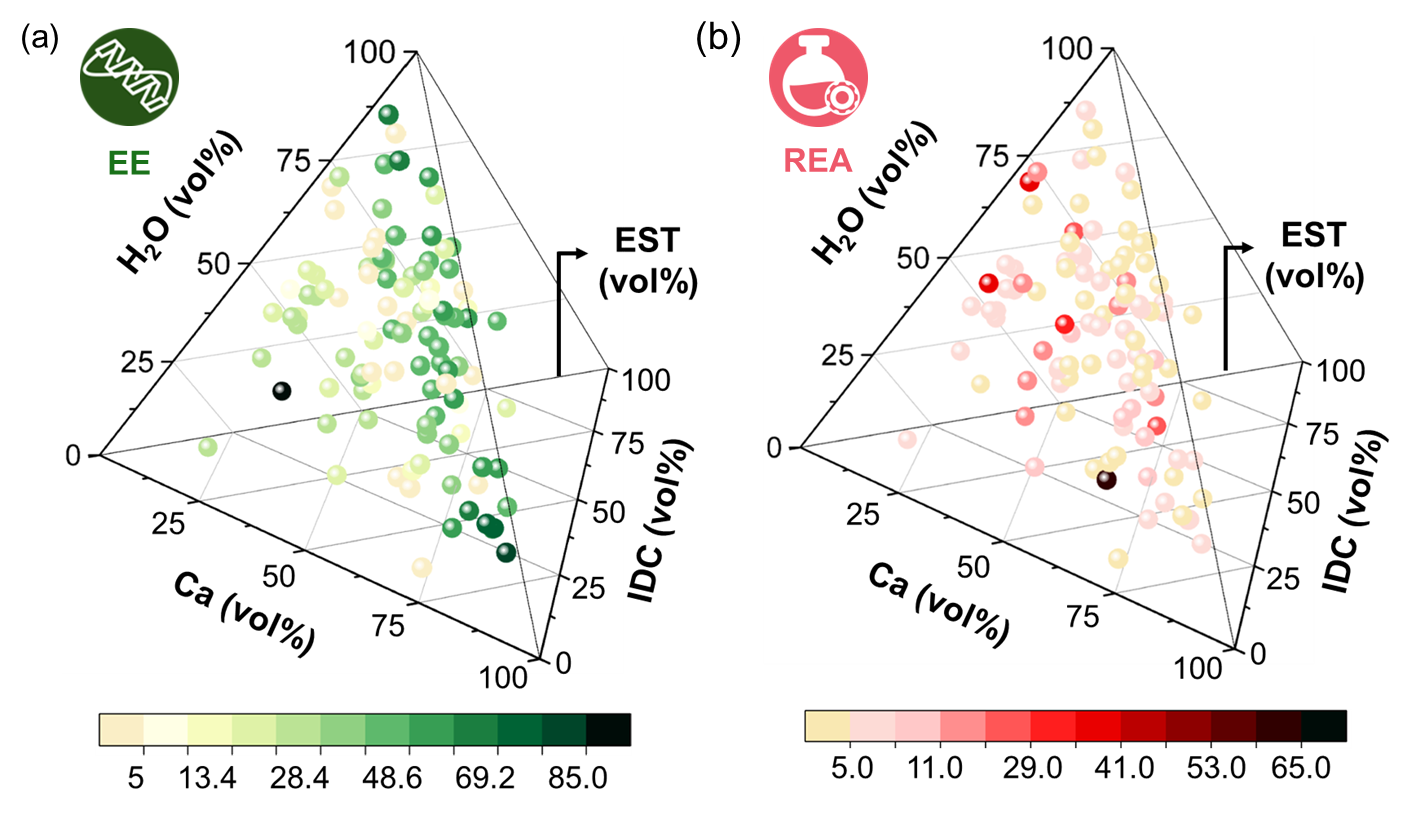


Figure S20 (a) Three-dimensional visualization showing both the seed data and the ninth iteration of EE (a, EE in %, green) and REA (b, REA in % red) values for EST/CaIDC. The colour scale and spot size represent the EE or REA values (%).


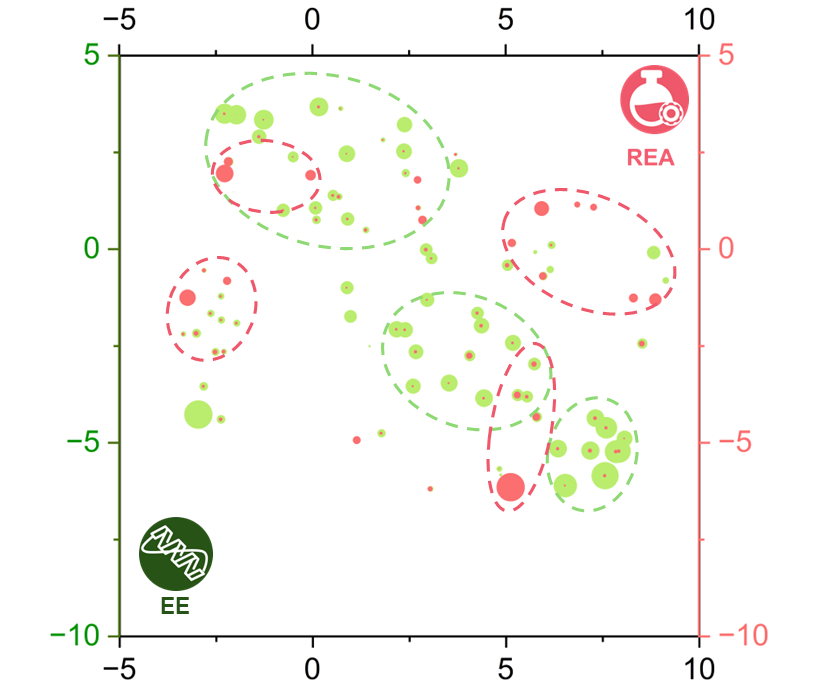


Figure S21 Two-dimensional t-SNE visualization showing all 105 distinct synthesis recipes for EST/CaIDC. Prior to dimension reduction, normalization is applied to all synthesis features. The size coding of the data points corresponds to the experimental values of EE (green) and REA (red), with larger spots indicating higher values. Optimal synthesis regions, conducive to achieving high-performance values, are outlined by circles with dashed lines.

**S3 Characterization of FBC78 and EC19**


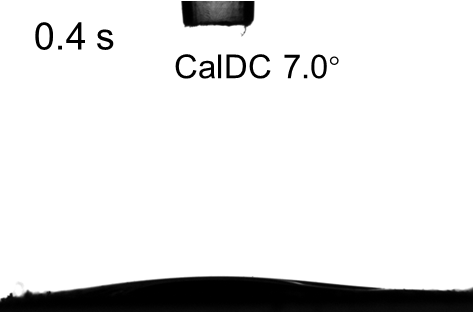


Figure S22 Contact angle images of CaIDC (Contact angle measurements of the samples prepared by evenly spreading them on glass slides secured with double-sided tape. The sessile drop method was employed using a Drop Shape Analyzer (Theta Flex Auto 2).

CaIDC exhibits superhydrophilicity, as evidenced by the rapid spreading and disappearance of the water droplet during contact angle measurement. This behavior further indicates its poor ability to retain surface-adsorbed biomolecules such as FBSA, highlighting the need for hydrophobic modification to improve surface interactions.


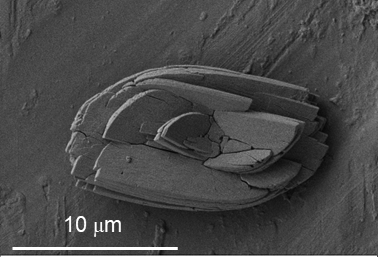


Figure S23 SEM images of the EC19.


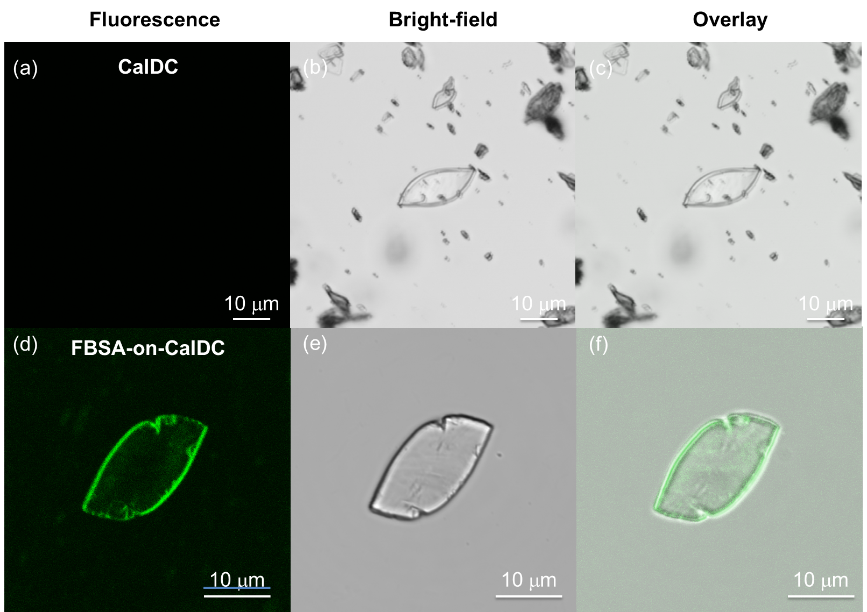


Figure S24 CLSM fluorescence images (a, d), bright-field images (b, e), and overlay images (c, f) of CaIDC and FBSA-on-CaIDC, respectively.

Under identical conditions, the CaIDC control sample exhibited no detectable fluorescence signal in CLSM images, confirming that the fluorescence observed originated exclusively from the FITC-labeled enzyme. Notably, literature reports indicate strong room-temperature phosphorescence properties of Ca(IDC)(CH_3_OH) and Ca(IDC)(CH_3_CH_2_OH), with maximum emission peaks around 552 nm and 566 nm, respectively, which are close to the emission wavelength of FITC.^[5]^ Additionally, the fluorescence signal of FBSA appeared exclusively at the edges of the CaIDC structure, and its intensity was significantly weaker than that of FBC78.


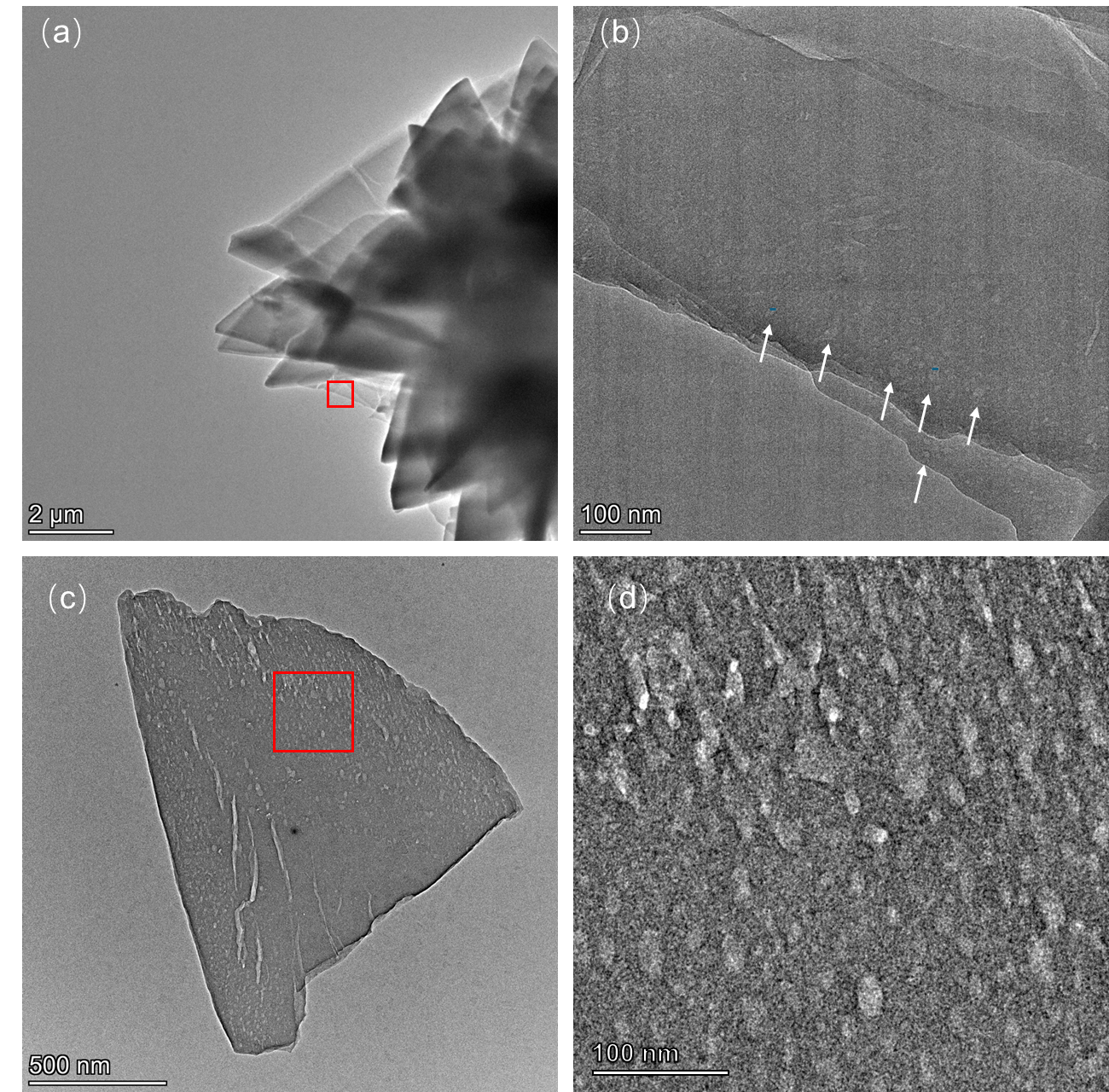


Figure S25 Transmission electron microscopy (TEM) images of EST@CaIDC composite after calcination at 300 °C for 2 h. (a) Low-magnification image showing overall morphology of the processed particles. (b) High-magnification image of the red-boxed region in (a), revealing nanoscale cavities (4–20 nm) attributed to the removal of embedded EST molecules or aggregates. (c) TEM image of fragmented EST@CaIDC crystal, (d) High-magnification image of the red-boxed region in (c), highlighting that most cavities are located near the crystal edge, consistent with CLSM results. These observations support the embedding of enzyme molecules within the CaIDC matrix rather than surface adsorption. The structure of EST@CaIDC was examined using the TEM on FEI Spectra 300 kV TEM.


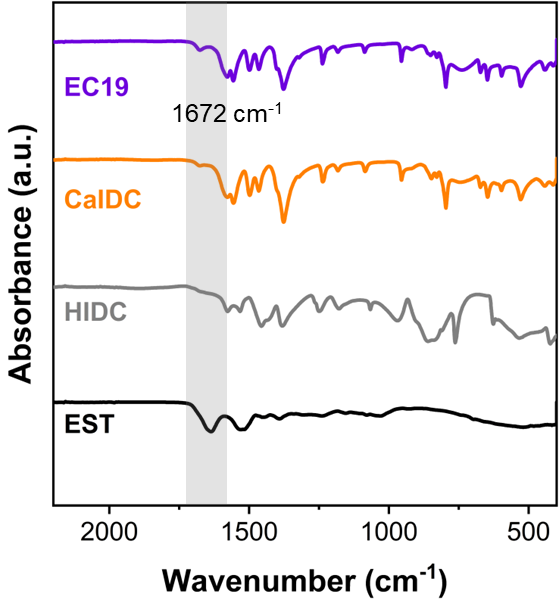


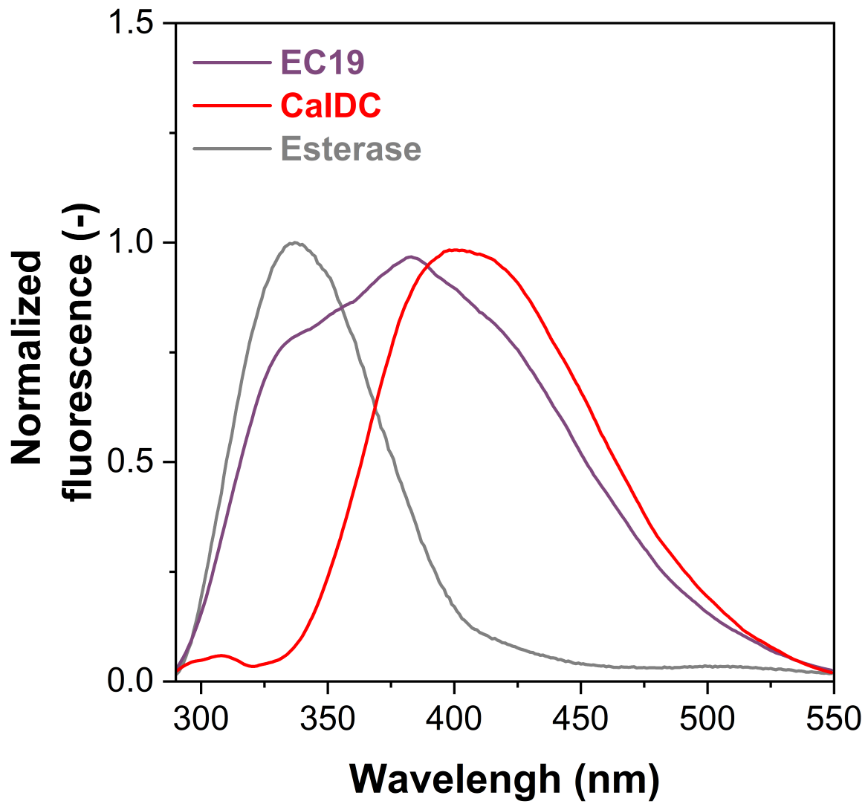
Figure S26 (a) ATR-FTIR spectra of EST (black), IDC (grey), CaIDC (orange), and EC19 (purple)

Figure S27 Fluorescence spectra of esterase (grey), CaIDC (red), and EC19 (purple) (λ_ex_ = 280 nm).

For CaIDC, two primary fluorescence emission peaks were observed at 383 nm and 422 nm, attributed to the $\pi\to\pi*$ transition of the ligand and the metal-ligand charge transfer, respectively.^[6]^ Following the encapsulation of EST, these peaks blue-shifted to 381 nm and 420 nm, indicating that the interaction between EST and CaIDC alters the coordination environment of the MOF's metal centers or organic ligands, thereby affecting the fluorescence properties.


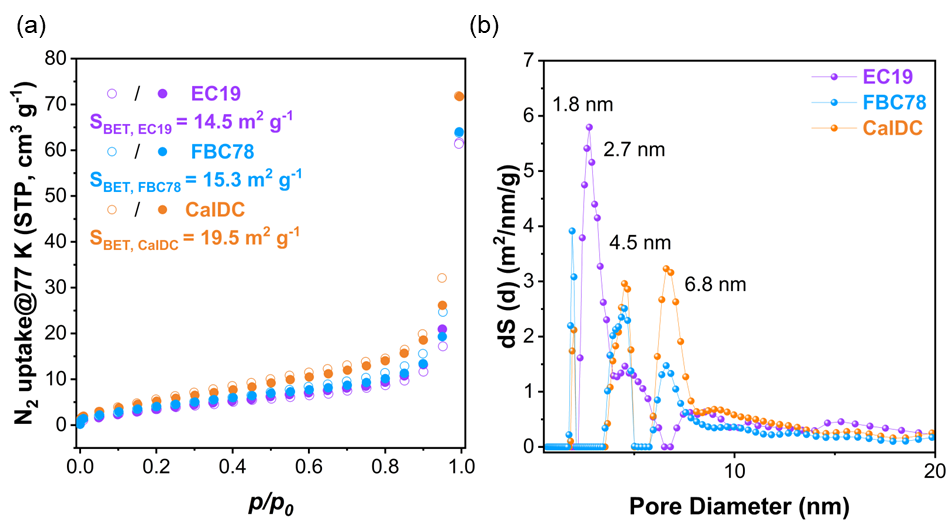


Figure S28 (a) 77 K N_2_ sorption isotherms of CaIDC (orange), FBC78 (blue), and EC19 (purple). Closed and open symbols represent adsorption and desorption isotherms, respectively. (d) NLDFT (NLDFT = non-linear density functional theory) pore size distribution curves of CaIDC (orange), FBC78 (blue), and EC19 (purple). Prior to sorption analysis, samples were degassed at 220 °C for 6 h.

**S4 Crystal Structure analysis**


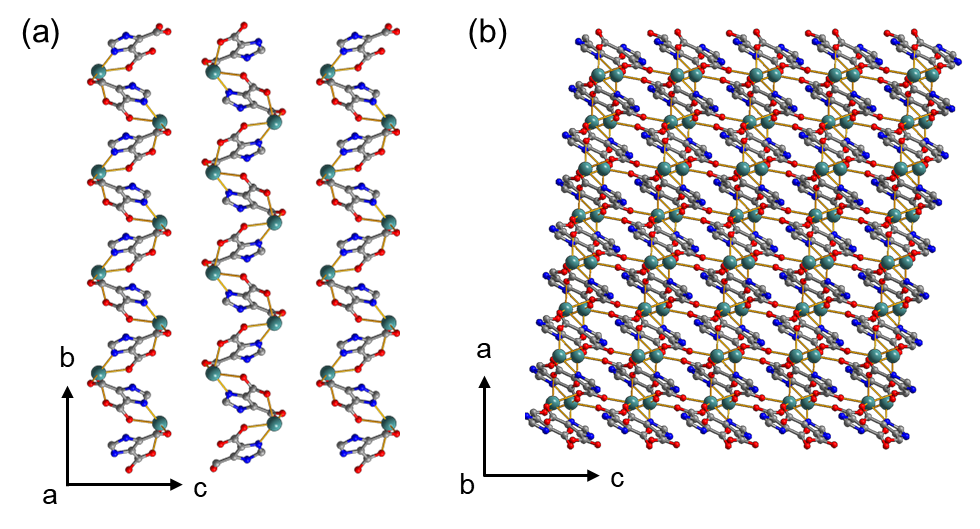


Figure S29 (a) Crystal structure of CaIDC ([Ca(IDC)(H₂O)₄]·H₂O) viewed along the a axis, corresponding to the (100) facet. (b) Crystal structure of CaIDC viewed along the b axis, corresponding to the (010) facet.

The (010) facet predominantly exposes Ca²⁺ sites, while the (100) facet primarily presents the N atoms at the 1- and 3-positions of the imidazole rings.

The asymmetric unit of CaIDC consists of one eight-coordinated Ca²⁺ ion and two IDC²⁻ ligands (Figure S29). As shown in Figure S29, the Ca²⁺ ion is coordinated by seven O atoms and one N atom, forming an eight-coordinate geometry.


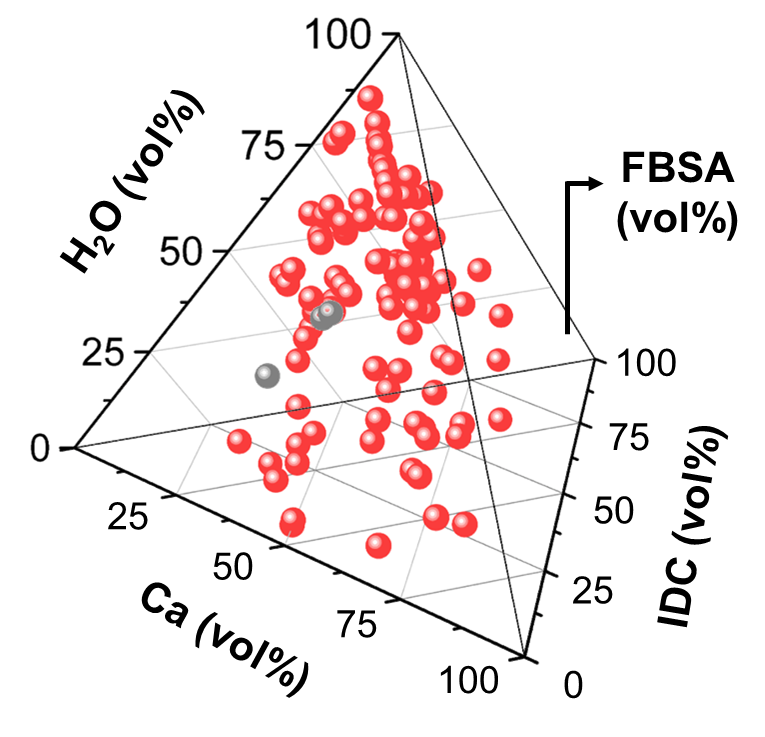


Figure S30 Three-dimensional visualization showing the crystallinity distribution of CaIDC. Amorphous products are shown in grey circles.


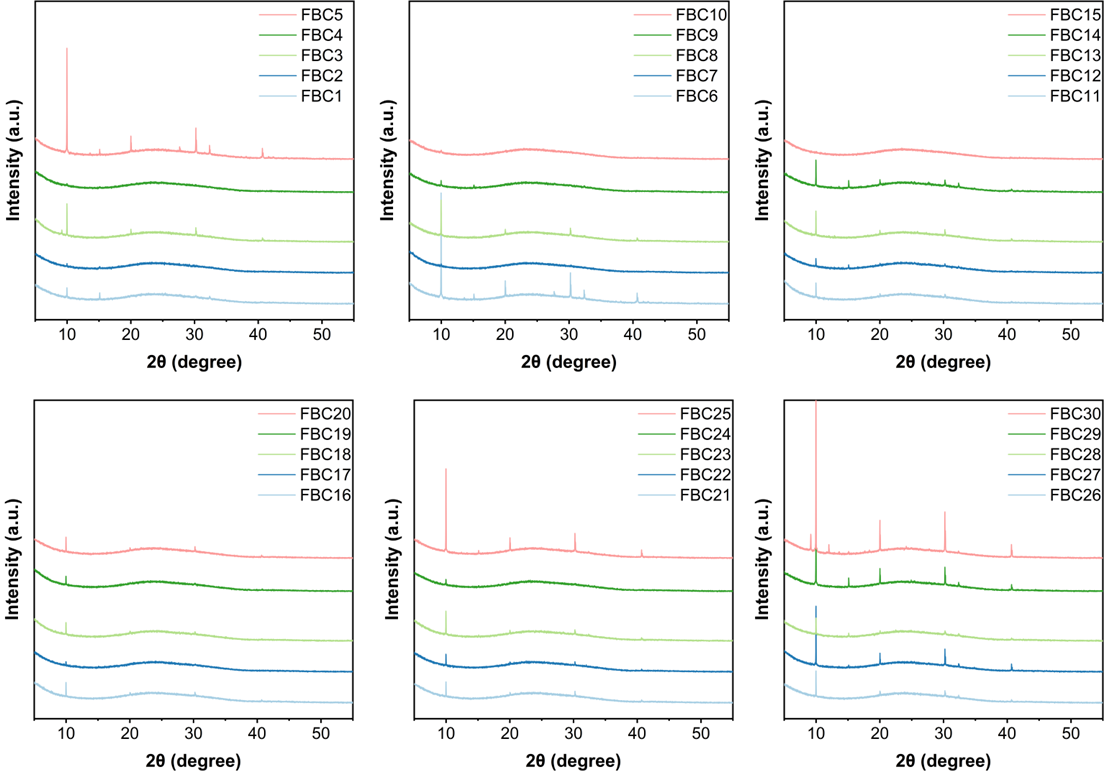


Figure S31 PXRD patterns of FBSA/CaIDC samples (FBC1-30) for machine learning.


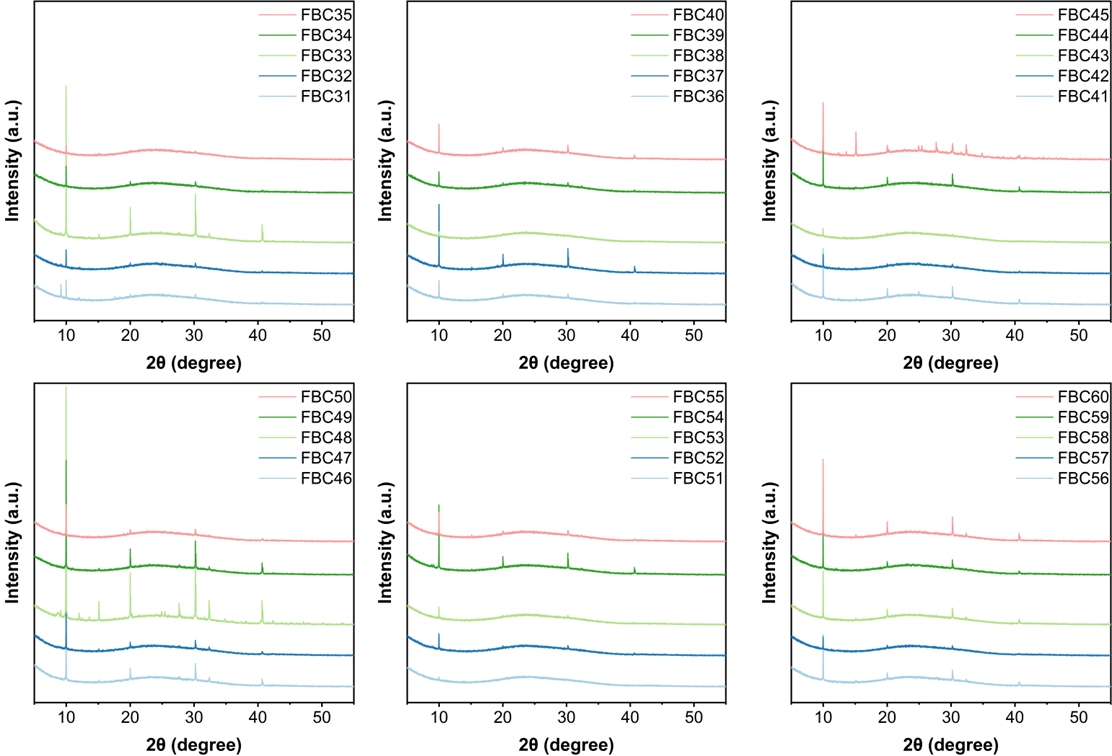


Figure S32 PXRD patterns of FBSA/CaIDC samples (FBC30-60) for machine learning.


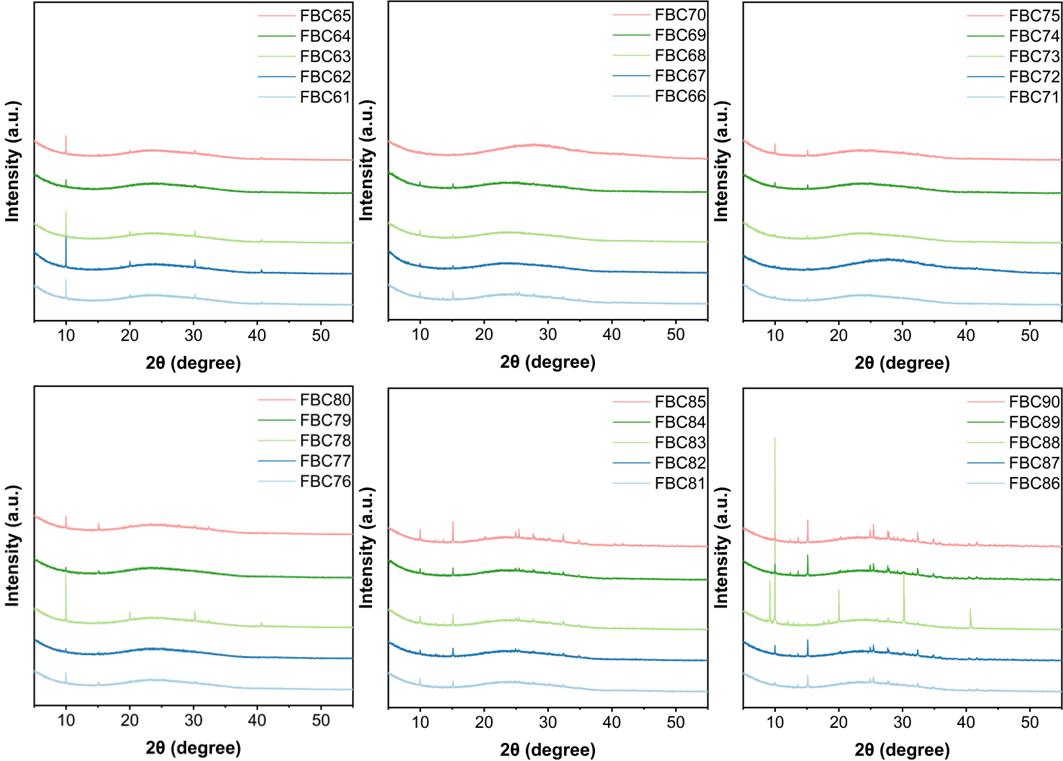


Figure S33 PXRD patterns of FBSA/CaIDC samples (FBC61-90) for machine learning.


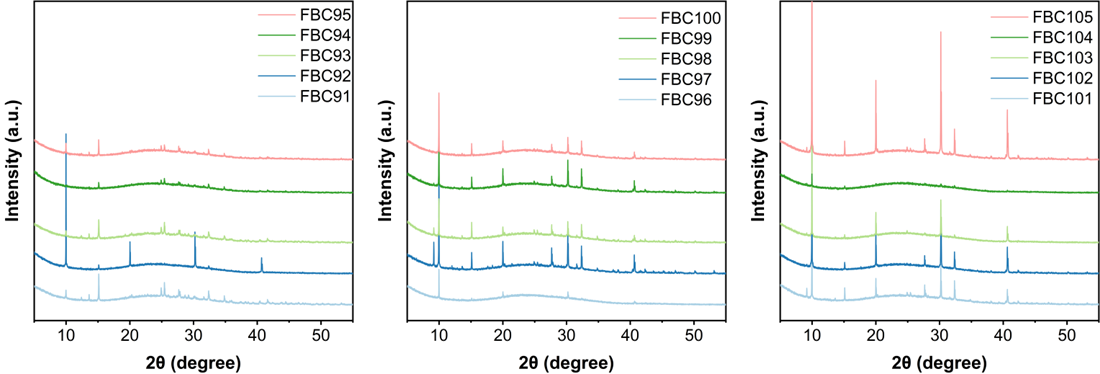


Figure S34 PXRD patterns of FBSA/CaIDC samples (FBC91-105) for machine learning.


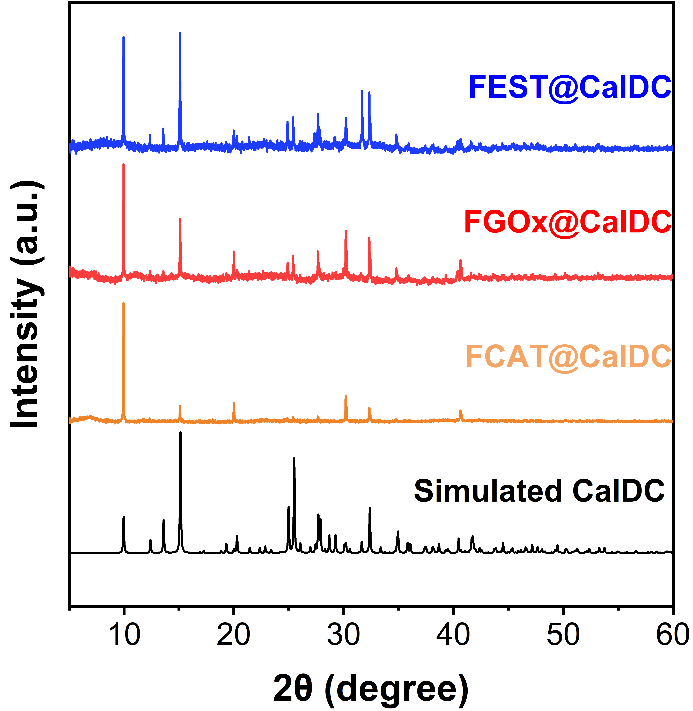


Figure S35 XRD patterns of FEST@CaIDC (blue), FGOx@CaIDC (red), and FCAT@CaIDC (orange).


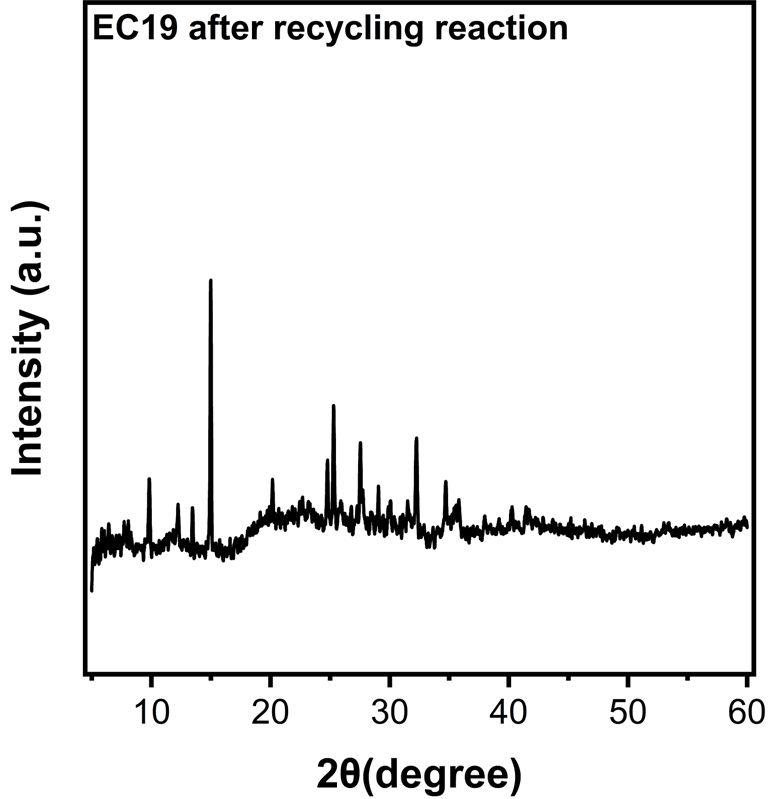


Figure S36 PXRD patterns of EC19 after recycling reaction.


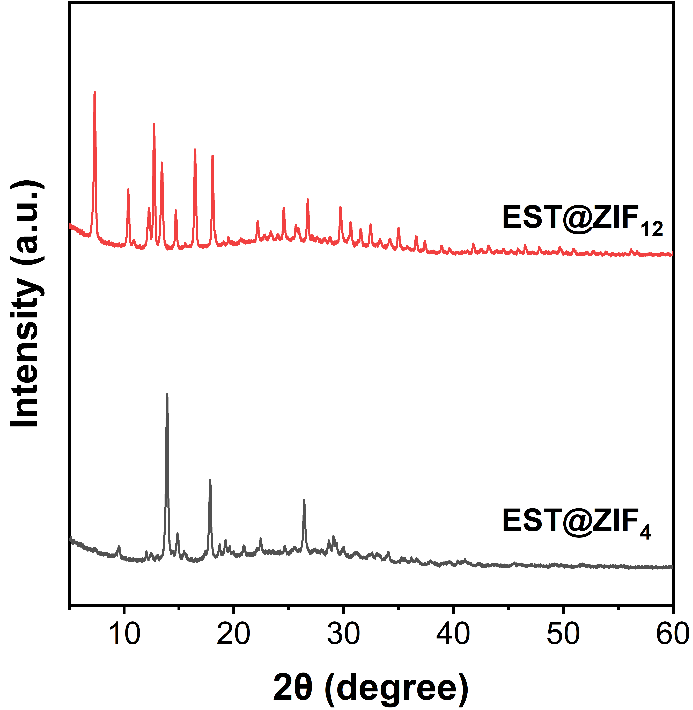


Figure S37 PXRD patterns of EST@ZIF_4_ and EST ZIF_12_. EST@ZIF_4_ crystallized as dia-Zn(mIM)_2_, while EST@ZIF_12_ crystallized as a mixed phase of dia-Zn(mIM)_2_ and sod-Zn(mIM)_2_.


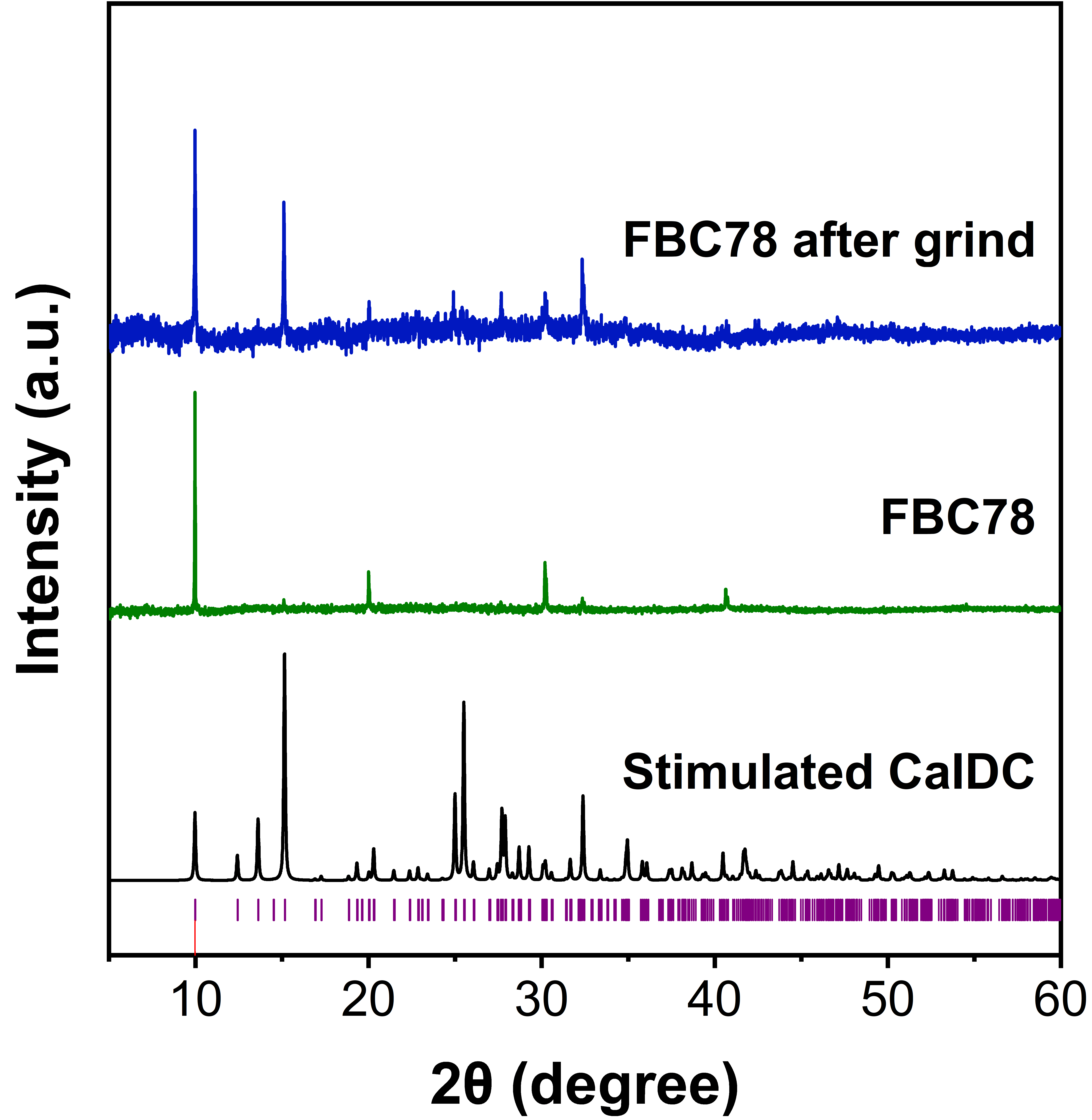


Figure S38 PXRD patterns of FBC78 before (green) and after (blue) grinding. FBC78 are preferentially growth on the [010] facet. After grinding, additional peaks corresponding to other crystal facets emerge in the PXRD measurement (blue), suggesting a disruption of the preferred orientation.

**S5 Formation kinetics of CaIDC and enzyme@CaIDC**


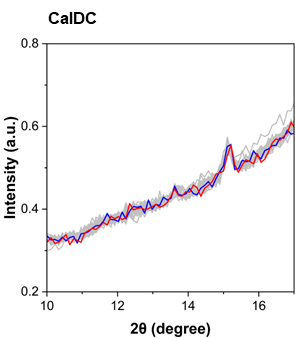


Figure S39 In-situ SAXRD data of CaIDC. The blue and red lines correspond to the first and last frames, respectively, during the 5-minute measurement period.


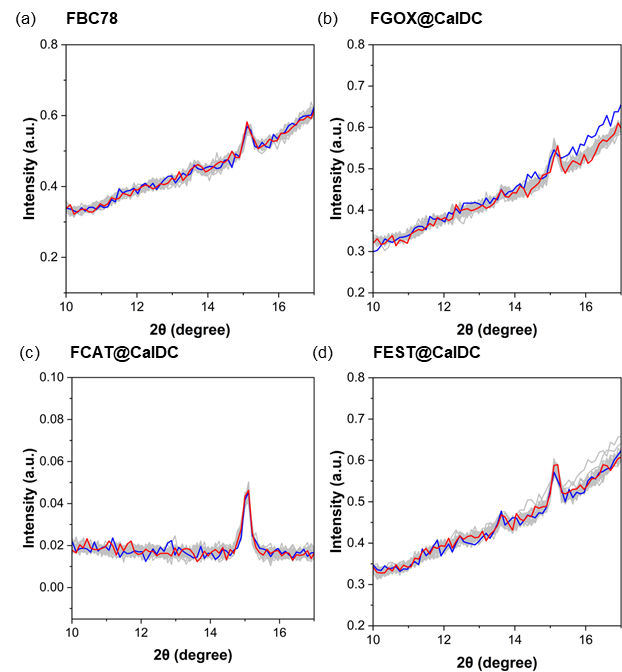


Figure S40 In-situ SAXRD data of FBC78 (a), FGOx/CaIDC (b), FCAT/CAIDC (c) and FEST/CaIDC (d). The blue and red lines correspond to the first and last frames, respectively, during the 5-minute measurement period.

According to our in-situ SAXRD results, the presence of enzymes, including FGOx, FEST, and FCAT, did not influence the crystallization process of CaIDC. Specifically, MOF crystals formed spontaneously within 1 minute under identical reaction conditions, regardless of whether enzymes were present or absent. This observation suggests that the crystallization of CaIDC follows the abiotic mineralization process. Unlike previously reported biomimetic mineralization processes, such as esterase-induced acceleration of MAF-6 formation^[7]^, where enzymes actively participate and promote crystal growth, our results indicate that the enzymes
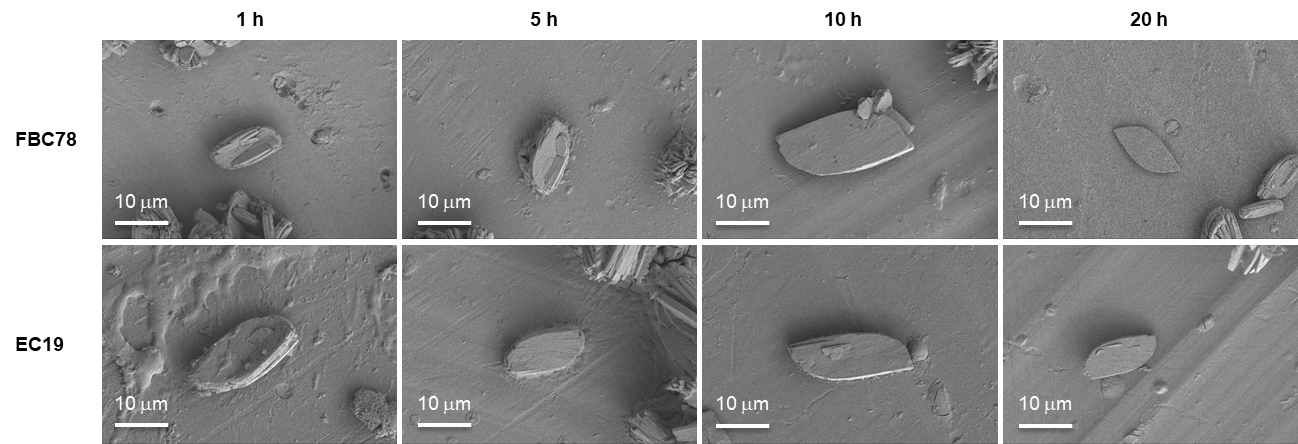
in the system remain inert during the crystallization process.

Figure S41 SEM images of FBC78 and EC19 synthesized at various reaction times (1 h, 5 h, 10 h, and 20 h).

**S6 Stability evaluation of FBC78 compared to FBSA@ZIF_4_ and FBSA@ZIF_12_**


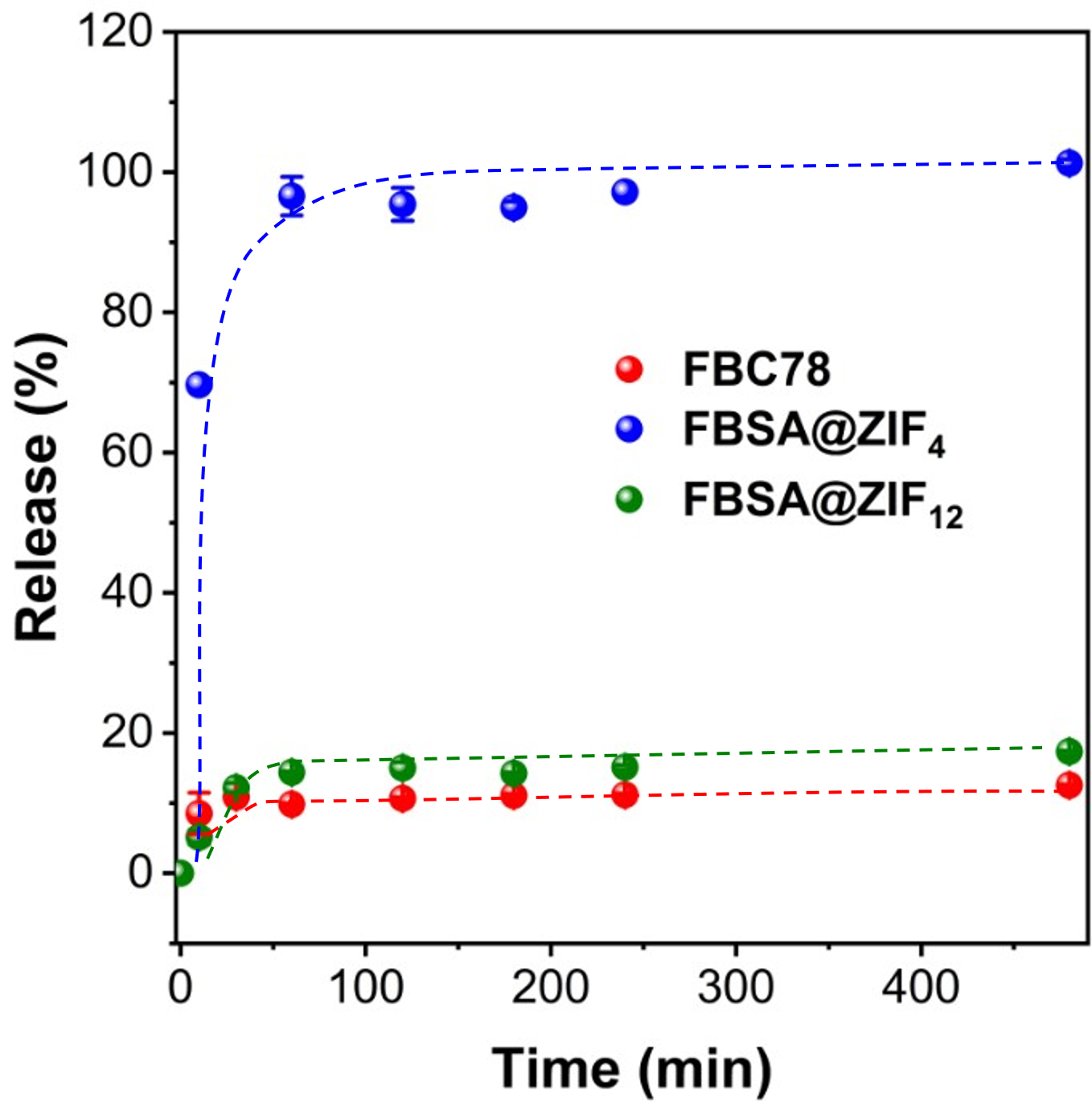


Figure S42 Kinetics of FBSA release from FBC78 (red), FBSA@ZIF_4_ (blue), and FBSA@ZIF_12_ (green) in pH 6.0, 0.01 M PB buffers.


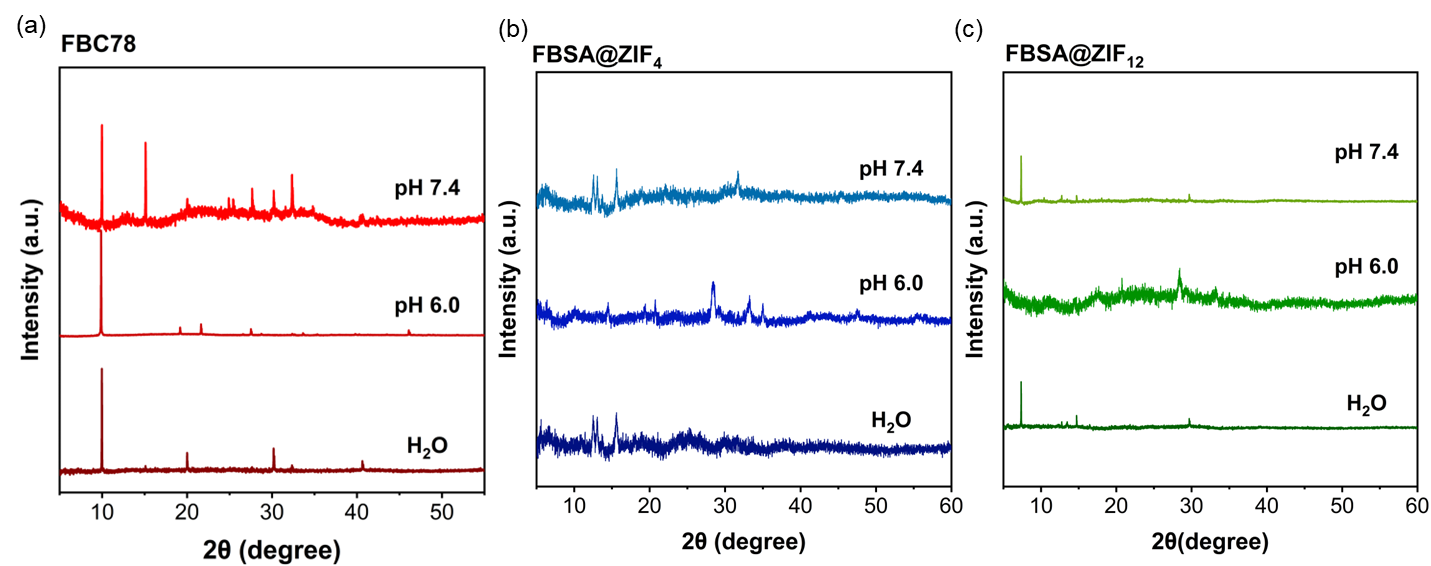


Figure S43 PXRD patterns of pristine and PB buffer exposed FBC78 (a, red), FBSA@ZIF_4_ (b, blue), and FBSA@ZIF_12_ (c, green).

After treatment in pH 7.4 PB buffer, additional crystal planes became visible in the PXRD pattern of FBC78, which initially displayed only the [010] peak. A similar effect was observed after mechanical grinding (Figure S38). These results suggest that FBC78 exhibits a preferential orientation along the [010] plane in its pristine state, likely due to growth conditions during synthesis. The exposure to PBS buffer or mechanical grinding disrupts this preferential alignment, leading to the exposure of other crystal planes.


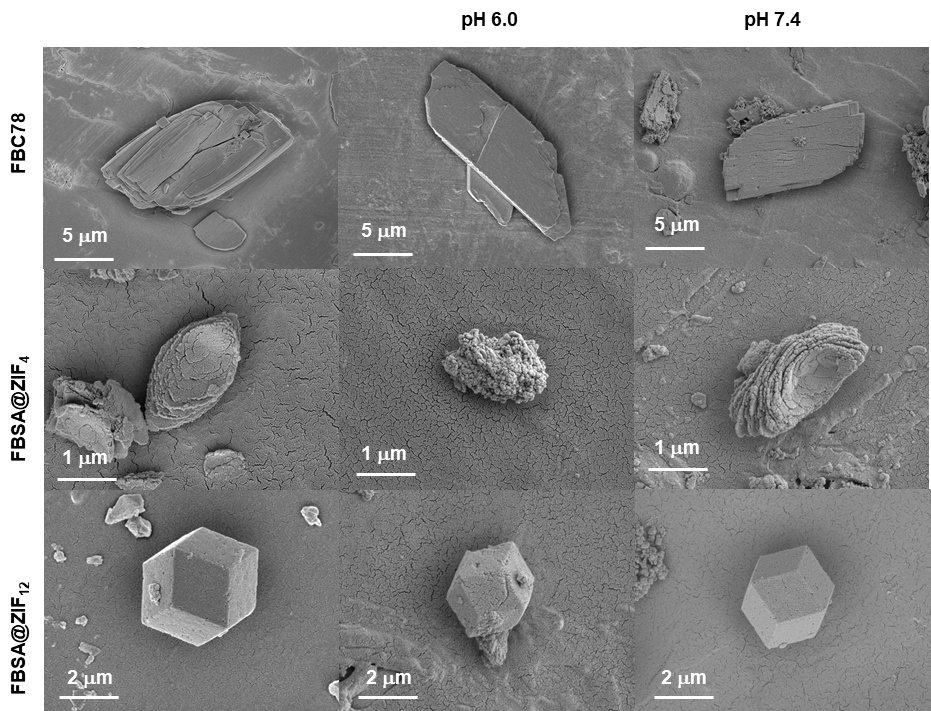


Figure S44 Upper row, from left: SEM images of the parent FBC78 and samples after immersion in PB buffers (pH 6.0 and 7.4). Middle row, from left: SEM images of the parent FBSA@ZIF_4_ and samples after immersion in PB buffers (pH 6.0 and 7.4). Bottom row, from left: SEM images of the parent FBSA@ZIF_12_ and samples after immersion PB buffers (pH 6.0 and 7.4).

After treatment in PB buffer at pH 6.0, FBSA@ZIF_4_ exhibited significant morphological changes, as observed in Figure S41. This further confirms its instability under this condition, likely due to the breakdown of the ZIF structure in the acidic environment.

**S7 Layer-by-layer growth of FBC78**


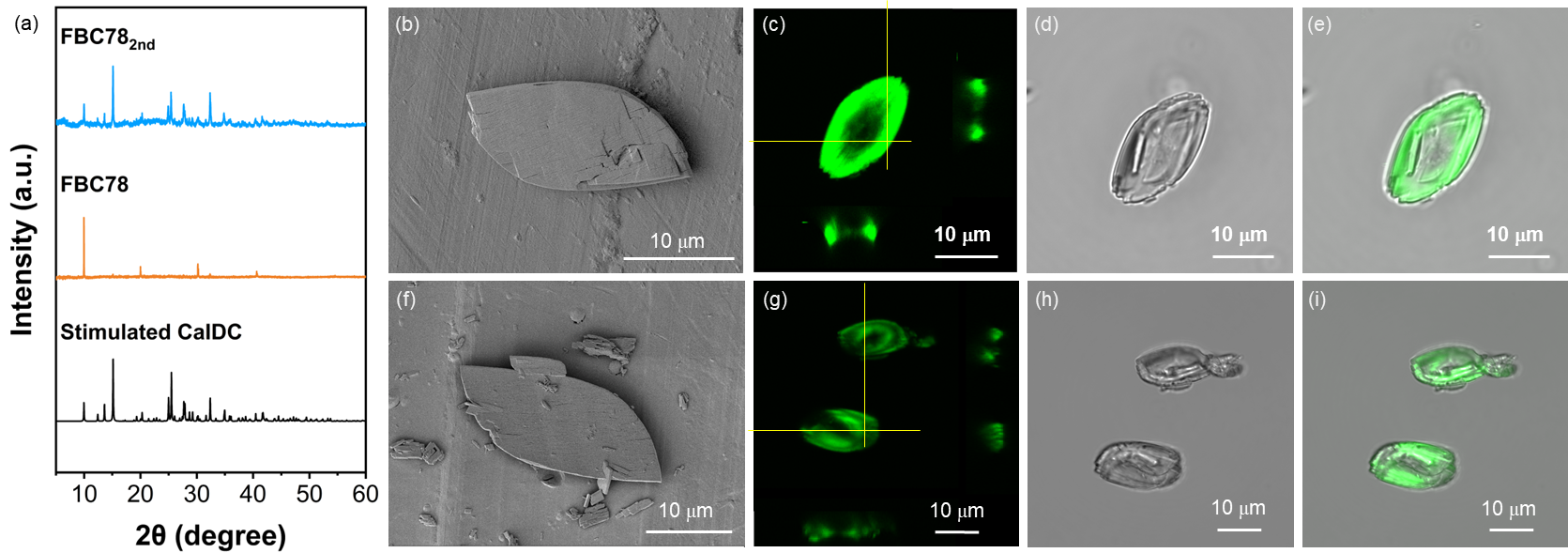


Figure S45 (a) PXRD patterns of FBC78 (orange) and FBC78_2nd_ (blue). Characterization of FBC78: (b) SEM image, (c) CLSM fluorescence image, (d) bright-field image, and (e) overlay image. Characterization of FBC78_2nd_: (f) SEM image, (g) CLSM fluorescence image, (h) bright-field image, and (i) overlay image.

**S8 Versatility of CaIDC as an enzyme immobilization platform**


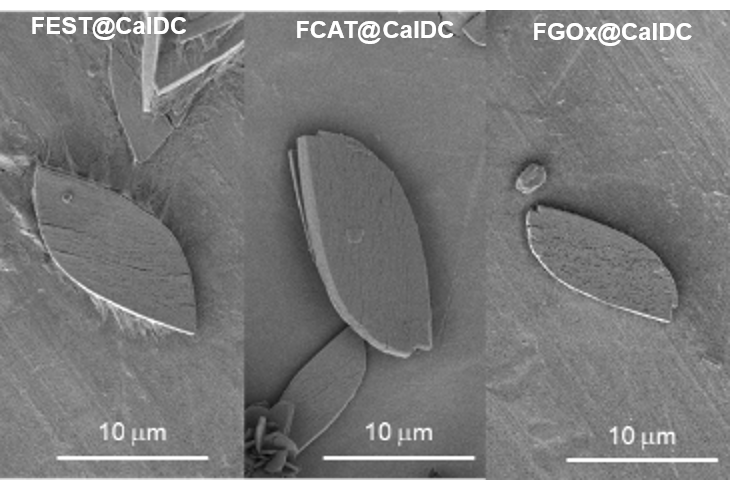


Figure S46 (a) From left to right: SEM image of FEST@CaIDC, FCAT@CaIDC, and FGOx@CaIDC.


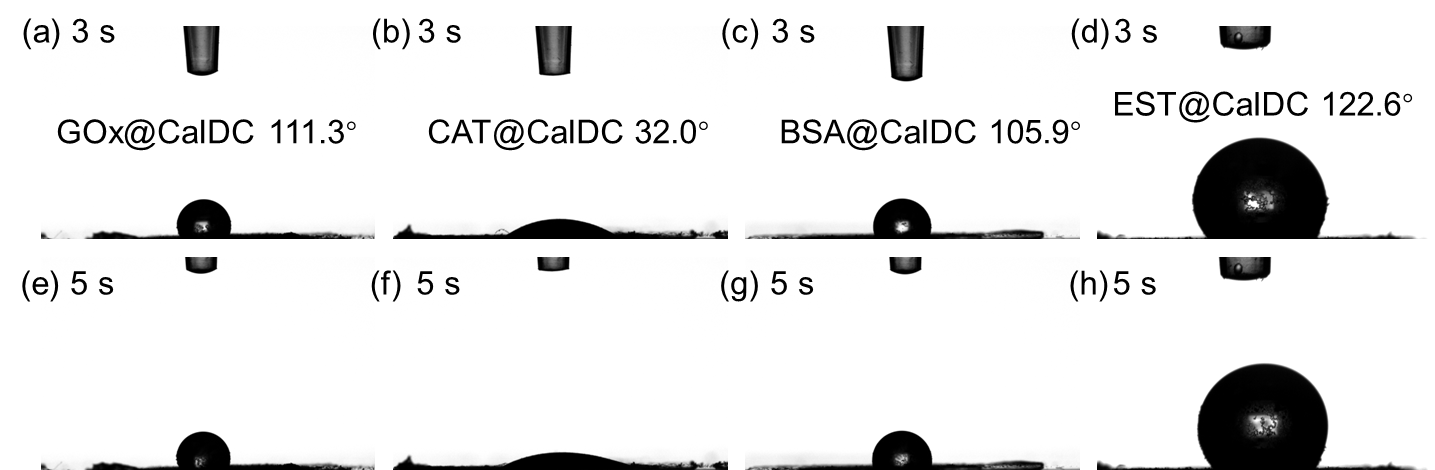


Figure S47 Contact angle measurements of various enzyme@CaIDC composites: (a, e) GOx@CaIDC, (b, f) CAT@CaIDC, (c, g) BSA@CaIDC, and (d, h) EST@CaIDC, recorded at 3 seconds (a–d) and 5 seconds (e–h) after water droplet deposition.

The contact angles for GOx@CaIDC, CAT@CaIDC, BSA@CaIDC, and EST@CaIDC were 111.3°, 32.0°, 105.9°, and 122.6°, respectively, and remained unchanged within the first 5 seconds (Figure S47). In contrast to the rapid spreading observed on pristine CaIDC, these results indicate that enzyme encapsulation significantly alters the surface wettability, diminishing the superhydrophilic nature of CaIDC.


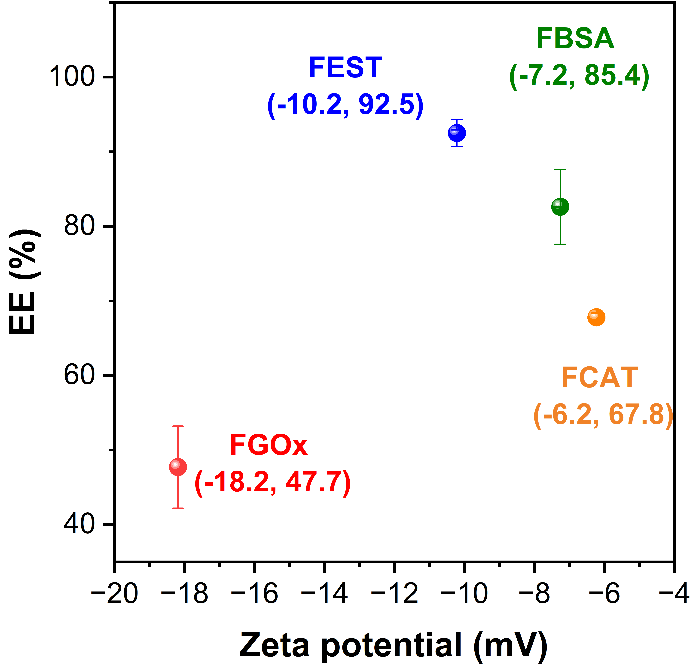


Figure S48 Zeta potentials and EE values for FEST (blue), FBSA (green), FGOx (red), and FCAT (orange).

**S9 Immobilization parameter summary for samples**

Table S1 EE (%), P_loading_ (wt%) and Yield (%) of Pure MOF for FBC78, FBC78_2nd_, FCAT@CaIDC, FGOx@CaIDC, FEST@CaIDC, FBSA@ZIF_4_, and FBSA@ZIF_12_.

| Sample | EE (%) | P_loading_ (wt%) | Yield_MOF_ (%) |
| --- | --- | --- | --- |
|  | Average | Average |  |
| FBC78 | 85.4 (1.7) | 16.1 (0.1) | 66.0 |
| FBC78_2nd_ | 48.3 (2.3) | 4.3 (0.2) | - |
| FCAT@CaIDC | 67.8 (0.0) | 0.2 (0.0) | 71.6 |
| FGOx@CaIDC | 47.7 (5.5) | 0.3 (0.03) | 85.8 |
| FEST@CaIDC | 92.5 (1.8) | 0.7 (0.01) | 76.8 |
| FBSA@ZIF_4_ | 38.3 (4.7) | 4.0 (0.5) | 50.9 |
| FBSA@ZIF_12_ | 72.8 (3.5) | 7.2 (0.3) | 49.6 |

Table S2 EE (%), REA (%) and P_loading_ (wt%) of EC19, EST@CaBDC, EST@ZIF_4_, and EST@ZIF_12_

| Sample | EE (%) | REA (%) | P_loading_ (wt%) |
| --- | --- | --- | --- |
|  | Average | Average | Average |
| EC19 | 28.7 (3.8) | 20.1 (4.8) | 4.2 (0.6) |
| EST@CaBDC | 43.8 (1.1) | 8.1 (3.2) | 0.94 (0.02) |
| EST@ZIF_4_ | 41.8 (6.6) | 2.7 (2.4) | 3.0 (0.5) |
| EST@ZIF_12_ | 20.0 (3.0) | 33.5 (2.6) | 0.7 (0.1) |

**S10 References**

[1] S. Goel, M. R. Feisal, G. I. Danmaliki, S. Yu, P. B. Liu, R. E. Bishop, F. G. West, P. M. Hwang, *Biochimica et Biophysica Acta (BBA) - Biomembranes* **2024**, 1866, 184281.

[2] J. T. Vivian, P. R. Callis, *Biophysical journal* **2001**, 80, 2093.

[3] C. Duy, J. Fitter, *Biophysical journal* **2006**, 90, 3704.

[4] W. Liang, S. Zheng, Y. Shu, J. Huang, *JACS Au* **2024**, 4, 3170.

[5] R.-H. Shi, Z.-Q. Long, F. Wang, L.-Z. Gong, X.-Y. Lin, G.-L. Zhuang, D.-F. Lu, *Chemical Engineering Journal* **2024**, 479, 147851.

[6] T. K. Pal, *Mater. Chem. Front.* **2023**, 7, 405.

[7] W. Liang, K. Flint, Y. Yao, J. Wu, L. Wang, C. Doonan, J. Huang, *J. Am. Chem. Soc.* **2023**, 145, 20365.
